# New Tripeptide Derivatives Asperripeptides A–C from Vietnamese Mangrove-Derived Fungus *Aspergillus terreus* LM.5.2

**DOI:** 10.3390/md20010077

**Published:** 2022-01-17

**Authors:** Elena V. Girich, Anton B. Rasin, Roman S. Popov, Ekaterina A. Yurchenko, Ekaterina A. Chingizova, Phan Thi Hoai Trinh, Ngo Thi Duy Ngoc, Mikhail V. Pivkin, Olesya I. Zhuravleva, Anton N. Yurchenko

**Affiliations:** 1 G.B. Elyakov Pacific Institute of Bioorganic Chemistry, Far Eastern Branch of the Russian Academy of Sciences, Prospect 100-Letiya Vladivostoka, 159, 690022 Vladivostok, Russia; ev.girich@piboc.dvo.ru (E.V.G.); rasin_ab@piboc.dvo.ru (A.B.R.); popov_rs@piboc.dvo.ru (R.S.P.); eyurch@piboc.dvo.ru (E.A.Y.); chingizova_ea@piboc.dvo.ru (E.A.C.); pivkin@piboc.dvo.ru (M.V.P.); 2 Department of Marine Biotechnology, Nhatrang Institute of Technology Research and Application, Vietnam Academy of Science and Technology, Nha Trang 650000, Vietnam; phanhoaitrinh@nitra.vast.vn (P.T.H.T.); ngoduyngoc@nitra.vast.vn (N.T.D.N.); 3 School of Natural Sciences, Far Eastern Federal University, 690922 Vladivostok, Russia

**Keywords:** marine-derived fungi, mangrove-derived fungi, secondary metabolites, diketopiperazines, tripeptide derivatives, cinnamic acid, sortase A, cytotoxicity

## Abstract

Three new tripeptide derivatives asterripeptides A–C (**1**–**3**) were isolated from Vietnamese mangrove-derived fungus *Aspergillus terreus* LM.5.2. Structures of isolated compounds were determined by a combination of NMR and ESIMS techniques. The absolute configurations of all stereocenters were determined using the Murfey’s method. The isolated compounds **1**–**3** contain a rare fungi cinnamic acid residue. The cytotoxicity of isolated compounds against several cancer cell lines and inhibition ability of sortase A from *Staphylococcus aureus* of asterripeptides A–C were investigated.

## 1. Introduction

The genus *Aspergillus* is a group of filamentous fungi, which today consists of more than 250 species. Moreover, this genus has been subdivided into several subgenera and sections based on morphology and genetics, such as subgenera *Nidulantes*, *Circumdati*, *Fumigati*, *Terrei*, *Candidi*, and so on. The section *Terrei* includes *Aspergillus terreus* along with its varieties, as well as *A. niveus*, *A. carneus*, *A. niveus* var. *indicus*, *A. allahabadii*, *A. ambiguous*, and *A. microcysticus* [1]. Fungi of the section *Terrei* are mainly characterized by the several metabolite classes such as butenolides [2] and other polyketides [3,4], sesquiterpenoids [5,6,7], meroterpenoids [8], and bisindolebenzoquinone alkaloids [9].

*Aspergillus terreus* is a cosmopolite species with high biotechnological potential [10]. For instance, *A. terreus* NIH2624 contains 28 polyketide synthase (PKS) genes, 22 non-ribosomal peptide synthetase (NRPS) genes, 1 hybrid PKS/NRPS gene, 2 PKS-like genes, and 15 NRPS-like genes [11]. Owing to the diversity of such synthase gene clusters, *A. terreus* produces a huge amount of secondary metabolites with very large structural variety. Marine environments have an additional effect on the structural features of *A. terreus* bioactive metabolites. Marine-derived *A. terreus* strains were described as producers of butyrolactones with anti-allergic, anti-inflammatory, and antiviral activities [2,12], an unusual antimicrobial *N*-phenyl-carbamic acid trimer [13] and a cytotoxic tetrapeptide [14].

Peptide derivatives are a very rare type of *A. terreus* metabolite [14,15]. Overall, low-weight marine fungal peptides are not as widespread as alkaloids, polyketides, and terpenoids, but they undoubtedly have pharmaceutical potential, thanks to their structural peculiarities and wide bioactivity spectrum [16]. Peptides of marine microorganisms, including fungi, can be biosynthetically produced by ribosomal and non-ribosomal peptide-synthase action, which can explain the presence of unique structural features, such as *D*-amino acids, *N*-terminally attached fatty acid chains, *N*- and *C*-methylated residues, *N*-formylated residues, heterocyclic elements, and glycosylated amino acids, as well as phosphorylated residues, among others [17]. For example, peptaibols—antibiotic active peptide-derivative compounds produced mainly by fungal species of the genus *Trichoderma* [18]. Moreover, there are cytotoxic, antiviral, antidiabetic, and anti-inflammatory peptides originating from marine fungi [19].

Previously, we reported a number of alkaloids, polyketides, and terpenoids from a Vietnamese mangrove-derived strain of *Aspergillus terreus* LM.5.2 [20].

**Figure 1:**
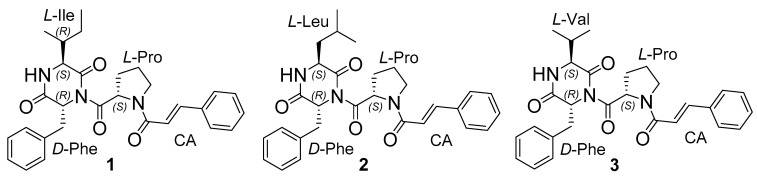
The structure of isolated **1**–**3** compounds.

Herein, we described the isolation and structural elucidation of three peptide-derivative secondary metabolites (Figure 1) of the same *Aspergillus terreus* LM.5.2 strain. Moreover, their cytotoxic activity against a number of malignant human cancer cells and non-malignant rat cardiomyocytes H9c2 cells, as well as inhibition of sortase A enzyme from *Staphylococcus aureus*, were investigated.

## 2. Results

The molecular formula of **1** was determined as C_29_H_33_N_3_O_4_ by the HRESIMS peak [M − H]^−^ at *m/z* 486.2399 (Figure S8, Supplementary Material), which was confirmed by the ^13^C NMR data. A thorough inspection of the ^1^H and ^13^C NMR data (Table 1, Figures S1–S3, Supplementary Material) of **1** identified the presence of two methyl groups (*δ*_H_ 0.85, 0.89; *δ*_C_ 12.1, 15.7), five methylene groups (*δ*_H_ 1.24, 1.40, 2.09, 2.14, 2.17, 2.50, 3.28, 3.33, 3.79, 3.90; *δ*_C_ 24.1, 24.4, 29.7, 38.5, 47.6), four aliphatic (*δ*_H_ 1.97, 2.54, 5.10, 5.22; *δ*_C_ 38.2, 58.2, 59.4, 61.7) and twelve aromatic (*δ*_H_ 6.73, 7.10 (2H), 7.29 (2H), 7.30, 7.35, 7.36 (2H), 7.51 (2H), 7.66; *δ*_C_ 117.9, 127.8, 127.9 (2C), 128.7 (2C), 128.8 (2C), 129.7, 130.5 (2C), 142.8) methine groups, two *sp*^2^-quarternary carbons (*δ*_C_ 135.0, 135.1), four amide carbonyl groups (*δ*_C_ 164.6, 168.2, 170.1, 174.7), and one NH-singlet (*δ*_H_ 5.57).

**Figure 2:**
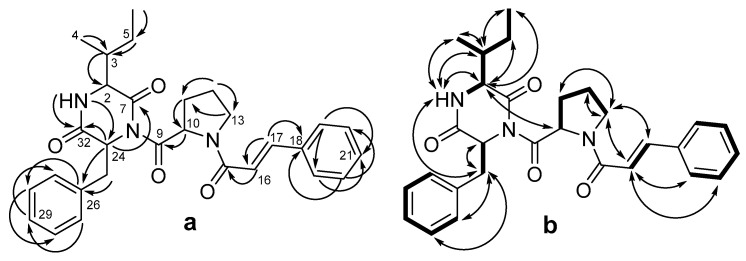
The key HMBC (**a**) and ^1^H-^1^H COSY and ROESY (**b**) correlation of **1**.

Detailed analysis of the HMBC and ^1^H-^1^H COSY and ROESY spectra (Figure 2, Figures S4–S7, Supplementary Material) led to the identification of three amino acid residues, including isoleucine (Ile), proline (Pro), and phenylalanine (Phe). The HMBC correlations (Figure 2a) from H-1 (*δ*_H_ 5.57) to C-24 (*δ*_C_ 58.2) and C-32 (*δ*_C_ 168.2), from H-2 (*δ*_H_ 2.54) to C-32, and from H-24 (*δ*_H_ 5.57) to C-7 indicated the presence of a diketopiperazine ring formed from residues of isoleucine and phenylalanine. The HMBC correlations (Figure 2a) from H-16 (*δ*_H_ 6.73) to C-15 (*δ*_C_ 164.6), C-17 (*δ*_C_ 142.8), and C-18 (*δ*_C_ 135.1); from H-17 (*δ*_H_ 6.73) to C-16 (*δ*_C_ 117.9), C-18, and C-19 (*δ*_C_ 127.9); from H-19 (*δ*_H_ 7.51) to C-17, C-21 (*δ*_C_ 129.7), and C-23(*δ*_C_ 127.9); from H-20 (*δ*_H_ 7.36) to C-22 (*δ*_C_ 128.8) and C-18; from H-21 (*δ*_H_ 7.35) to C-19 and C-23; and from H-23 (*δ*_H_ 7.51) to C-21, C-19, and C-17, along with the vicinal coupling constant (^3^J_H16-H17_ = 15.5 Hz) and MS/MS data (the peak at *m/z* 200.1061 ([C_13_H_14_NO]^−^) corresponding to cinnamic acid (CA) with Pro fragment, and the peak at *m/z* 131.0487 ([C_9_H_7_O]^−^) corresponding to CA), indicated the presence of a *trans*-cinnamic acid residue (Figure S8, Supplementary Material). This moiety was bound to the proline residue through the carboxyl group of the former and the amino group of the latter, as the ROESY spectrum contained correlations between H-13a (*δ*_H_ 3.93) and H-16 (*δ*_H_ 6.73), H-13b (*δ*_H_ 3.79), and H-16, as well as between H-13a/b and H-17 (*δ*_H_ 7.66). Thus, the planar structure of compound **1** was determined.

The absolute configurations of all stereocenters in **1** were established by Marfey’s method [21]. Analysis of *L*-FDAA derivatives of amino acid residues obtained by acid hydrolysis of compound **1** showed them to be derivatives of *L*-Ile, *L*-Pro, and *D*-Phe standard samples (Figures S25–S28, Supplementary Material). Thus, the configurations of chiral centers at C-2, C-10, and C-24 were determined as 2*S*, 10*S*, and 24*R*, respectively. Compound **1** was named asterripeptide A.

The molecular formula of **2** was suggested as C_29_H_33_N_3_O_4_ by the HRESIMS data (the [M + Na]^+^ peak at *m/z* 439.2367 (Figure S16, Supplementary Material)) and was confirmed by ^13^C NMR spectra (Figure S10, Supplementary Material).

**Figure 3:**
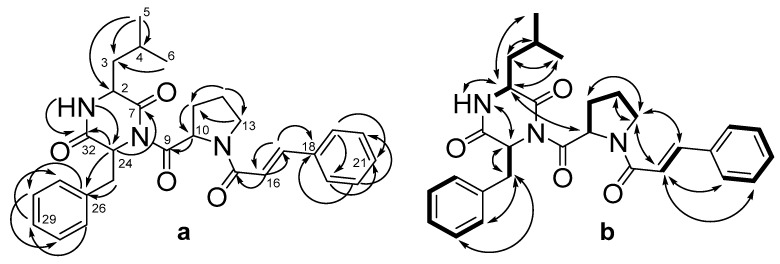
The key HMBC (**a**) and ^1^H-^1^H COSY and ROESY (**b**) correlations of **2**.

A detailed ^1^H and ^13^C NMR data comparison of **1** and **2** revealed a very close similarity with the exception of the signals in diketopiperazine moiety, namely C-3 (*δ*_C_ 40.7), C-4 (*δ*_C_ 24.4), C-5 (*δ*_C_ 23.1), and C-6 (*δ*_C_ 20.7), and a splitting pattern of the protons on these carbons (Table 2, Figures S9–S11, Supplementary Material). These data, along with HMBC correlations (Figure 3a, Figure S13, Supplementary Material) from H-4 (*δ*_H_ 2.07) to C-2 (*δ*_C_ 52.6) and C-3, from H-5 (*δ*_H_ 0.88) to C-2 and C-3, as well as from H-6 (*δ*_H_ 0.70) to C-3, suggest the presence of leucine residue (Leu) in the diketopiperazine ring of **2**, instead of Ile in **1**. Thus, compound **2** was determined to be an isomer of **1**.

The absolute configurations of all stereocenters in **2** were established by Marfey’s method [21]. Analysis of *L*-FDAA derivatives of amino acid residues obtained by acid hydrolysis of compound **2** showed them to be derivatives of *L*-Leu, *L*-Pro, and *D*-Phe standard samples (Figures S29–S32, Supplementary Material). Thus, the configurations of chiral centers at C-2, C-10, and C-24 were determined to be 2*S*, 10*S*, and 24*R* respectively. Compound **2** was named asterripeptide B.

The HRESIMS peak at *m/z* 472.2238 [M − H]^−^ (Figure S24, Supplementary Material) obtained for compound **3** corresponded with the molecular formula C_28_H_31_O_4_N_3_, which was confirmed by NMR data (Figures S17–S19, Supplementary Material).

**Figure 4:**
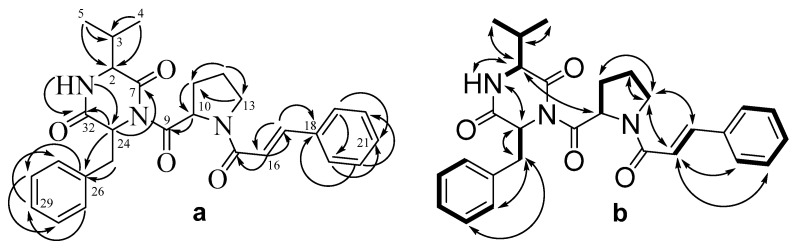
The key HMBC (**a**) and ^1^H-^1^H COSY and ROESY (**b**) correlations of **3**.

Detailed analysis of HMBC and ^1^H-^1^H COSY and ROESY spectra (Table 3, Figure 4, Figures S21–S23, Supplementary Material) showed the molecule of compound **3** to be mostly structurally close to asterripeptide A (**1**), except for the signals in diketopiperazine moiety. The HMBC correlations from H-3 (*δ*_H_ 2.31) to C-2 (*δ*_C_ 58.0), C-4 (*δ*_C_ 15.8), and C-5 (*δ*_C_ 18.9); from H-4 (*δ*_H_ 0.93) to C-2, C-3 (*δ*_C_ 31.7), and C-5; as well as from H-5 (*δ*_H_ 0.90) to C-2, C-3, and C-4 showed the presence of isopropyl-substitute in **3** instead of 2-butyl-substitute in **1**. Thus, the diketopiperazine fragment of **3** includes valine residue (Val) instead of Ile in **1**.

The absolute configurations of all stereocenters in **3** were established in the same way as in compounds **1** and **2**, by Marfey’s method. An analysis of *L*-FDAA derivatives of amino acid residues obtained by acid hydrolysis of compound **3** showed them to be derivatives of *L*-Val, *L*-Pro, and *D*-Phe standard samples (Figures S33–S36, Supplementary Material). Thereby, the configurations of chiral centers at C-2, C-10, and C-24 were determined to be 2*S*, 10*S,* and 24*R*, respectively. Compound **3** was named asterripeptide C.

It should be noted that cinnamic acid and its derivatives are widespread among plant metabolites [22,23]. However, these compounds were never reported as fragments of any fungal metabolites. Thus, this is the very first report of cinnamic acid containing compounds from marine microfilamentous fungus. We suggest that cinnamic acid was produced in the fungus from phenylalanine via phenylalanine-ammonia-lyase enzyme action, similar to its biosynthesis in plants [24]. A similar enzyme was earlier reported for *Aspergillus orizae* [25].

### Bioassays

The cytotoxic activities of isolated compounds **1**–**3** are presented in Table 4. Each one showed a weak cytotoxic activity toward non-malignant cardiomyocyte H9c2 cells as well as human colorectal DLD-1 and breast cancer MCF-7 cells. The cytotoxic effect of compounds **1**–**3** against human prostate cancer PC-3 cells was not great either, but it was observed at lesser concentrations.

Asterripeptide A (**1**) half-maximal decreased the viability of MCF-7 and DLD-1 cells at 96.8 and 87.7 µM, respectively, whereas it decreased the viability of PC-3 cells at the concentration of 64.6 µM. Asterripeptide B (**2**) half-maximally decreased the viability of MCF-7 and DLD-1 cells at a concentration larger than 100 µM, whereas it decreased the viability of PC-3 cells at the concentration of 75.5 µM. Finally, asterripeptide C (**3**) decreased the viability of MCF-7 and DLD-1 cells by 50% at 96.6 and 84.9 µM, respectively, whereas it decreased the viability of PC-3 cells at the concentration of 58.3 µM. Thus, cytotoxicity of asterripeptides A–C (**1**–**3**) was not great, but some selectivity of these compounds for prostate cancer cells may be of particular interest.

**Figure 5:**
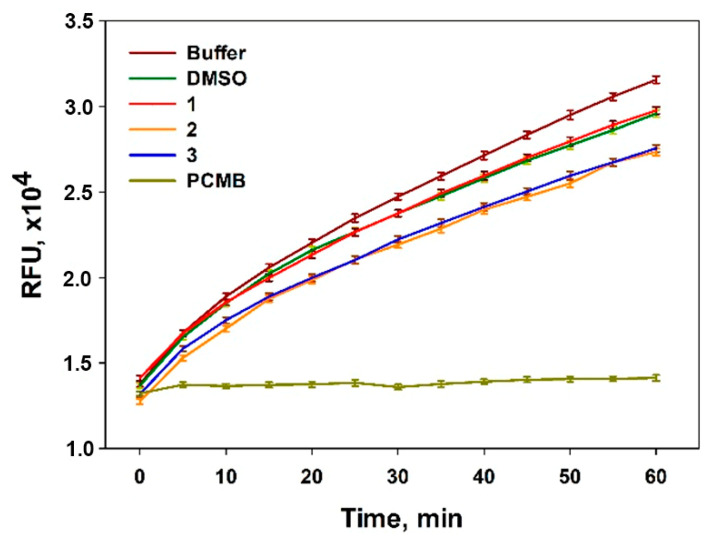
Effects of asterripeptides A–C (**1**–**3**) on the enzymatic activity of sortase A. Reaction kinetics of sortase A in the presence of isolated compounds (80 μM) and selective inhibitor 4-(hydroxymercuri)benzoic acid (PCMB) are at the same level of substrate concentration. DMSO (0.8%) was used as a vehicle.

To determine antibacterial properties, the possibility of isolated compounds inhibiting enzymatic activity of the sortase A was investigated. The membrane-associated sortase A enzyme (EC 3.4.22.70) is one of the key enzymes for *Staphylococcus aureus* virulence, and its inhibitors are considered as potential anti-bacterial agents [26]. Thus, asterripeptides B (**2**) and C (**3**) inhibited sortase A activity by more than 20% at the concentration of 80 µM and asterripeptide A (**1**) was inactive in this assay (Figure 5).

Earlier bicyclic peptides with Leu-Pro-Pro motive were identified as potent and selective inhibitors of sortase A via binding to the enzyme’s active site, owing to structural mimicking of the Leu-Pro-Xaa-Thr-Gly region of natural substrate of sortase A [27]. Presumably, the presence of the terminal ethyl group in isoLeu is the main reason for the inactivity of asterripeptide A, and the Leu-Pro fragment in the structure of asterripeptide B (**2**) as well as the Val-Pro region in the structure of asterripeptide C (**3**) play a role in their sortase A inhibitory effect. Thus, asterripeptides B and C are worthy of note as promising anti-Staphylococcal agents, and their antibacterial activity will be investigated in detail in the future.

## 3. Materials and Methods

### 3.1. General Experimental Procedures

NMR spectra were recorded in CDCl_3_ on a Bruker DPX-500 (Bruker BioSpin GmbH, Rheinstetten, Germany) and a Bruker DRX-700 (Bruker BioSpin GmbH, Rheinstetten, Germany) spectrometer, using TMS as an internal standard. HRESIMS spectra were measured on a Maxis Impact mass spectrometer (Bruker Daltonics GmbH, Rheinstetten, Germany).

Low-pressure liquid column chromatography was performed using silica gel (50/100 μm, Imid Ltd., Krasnodar, Russia). Plates (4.5 cm × 6.0 cm) precoated with silica gel (5–17 μm, Imid Ltd.) were used for thin-layer chromatography. Preparative HPLC was carried out on a Shimadzu LC-20 chromatograph (Shimadzu USA Manufacturing, Canby, OR, USA) using a YMC ODS-AM (YMC Co., Ishikawa, Japan) (5 µm, 10 mm × 250 mm) and YMC SIL (YMC Co., Ishikawa, Japan) (5 µm, 10 mm × 250 mm) columns with a Shimadzu RID-20A refractometer (Shimadzu Corporation, Kyoto, Japan). Analysis of amino acids’ stereo configurations was performed on an Agilent 1100 chromatograph using YMC C-18 Pro column (YMC Co., Ishikawa, Japan) (5 µm, 4.6 mm × 250 mm).

### 3.2. Fungal Strain

The strain was isolated from mangrove tree leaves *Kandelia candel* (coast of Khanh Hoa province, Vietnam, South China Sea) on malt extract agar, and identified based on morphological and molecular features. For DNA extraction, the culture was grown on malt extract agar under 28 °C for 7 days. DNA extraction was performed with the HiPurA^TM^ Plant DNA Isolation kit (CTAB Method) (HiMedia Laboratories Pvt. Ltd., Mumbai, India) according to the manufacturer’s instructions. Fragments containing the ITS regions were amplified using ITS1 and ITS4 primers. The newly obtained sequences were checked visually and compared to available sequences in the GenBank database (https://www.ncbi.nlm.nih.gov/genbank, accessed on 16 December 2021). According to BLAST analysis of the ITS1–5.8S–ITS2 sequence, the strain LM.1.5 had 98.06% similarity with *Aspergillus terreus* DTO 403-C9 (sequence number in GenBank database—MT316343.1). The sequences were deposited in the GenBank nucleotide sequence database under MN788658.1.

### 3.3. Cultivation of Fungus

The fungus was cultured at 28 °C for three weeks in 40 × 500 mL Erlenmeyer flasks, each containing rice (20.0 g), yeast extract (20.0 mg), KH_2_PO_4_ (10 mg), and natural sea water (40 mL).

### 3.4. Extraction and Isolation

The fungal mycelia with the medium were extracted for 24 h with 12 L of EtOAc. Evaporation of the solvent under reduced pressure gave a dark red-brown oil (6.50 g), to which 250 mL H_2_O–EtOH (4:1) was added, and the mixture was thoroughly stirred to yield a suspension. It was extracted successively with hexane (100 mL × 3), EtOAc (150 mL × 3), and *n*-BuOH (150 mL × 2). After evaporation of the EtOAc layer, the residual material (4.00 g) was passed through a silica gel column (11 cm × 4 cm, 75 g), which was eluted first with n-hexane (1.0 L), followed by a step gradient from 5% to 100% EtOAc in *n*-hexane (total volume 25 L). Fractions of 250 mL each were collected and combined based on the TLC results (silica gel and toluene–2-propanol, 6:1 and 3:1, *v*/*v*).

The AT-1-40 (470.08 mg) fraction eluted by the *n*-hexan–EtOAc system (85:15) was separated by an LH-20 column (80 cm × 2 cm, 50 g) with CHCI_3_ to yield subfraction AT-27-1 (50.62 mg). Subfraction AT-27-1 was purified by HPLC on a YMC-SIL column, eluting with *n*-hexan–EtOAc (50:50) to yield compounds **1** (7.94 mg) and **2** (4.63 mg). The AT-101-69 (154 mg) fraction eluted by *n*-hexan–EtOAc system (75:25) was separated by LH-20 column (80 cm × 2 cm, 50 g) with CHCI_3_ to yield subfraction AT-103-13 (33.64 mg). The subfraction AT-103-13 was purified by HPLC on a YMC ODS-AM with MeCN–H_2_O (75:25) to yield **3** (3.41 mg).

Asterripeptide A (**1**): colorless oil; ^1^H and ^13^C NMR data, see Table 1, Figures S1–S7; HRESIMS [M − H]^−^ 486.2473 (calc. for C_29_H_32_N_3_O_4_, 486.2471).

Asterripeptide B (**2**): colorless oil; ^1^H and ^13^C NMR data, see Table 2, Figures S9–S15; HRESIMS [M + Na]^+^ 439. 2367 (calc. for C_29_H_33_N_2_O_5_Na, 510.2369).

Asterripeptide C (**3**): colorless oil; ^1^H and ^13^C NMR data, see Table 3, Figures S17–S23; HRESIMS [M + Na]^+^ 496.2203 (calc. for C_28_H_31_N_3_O_4_Na, 496.2207).

### 3.5. Stereo Configuration Analysis of Amino Acids in Compounds ***1**–**3***

The compounds (0.2 mg each) were placed in glass ampoules and dissolved in 6N HCl (0.4 mL). Solutions in ampoules were frozen in liquid nitrogen, then vacuumed, sealed, and heated at 105 °C for 24 h. Then, the cooled reaction mixture was diluted by distilled water and concentrated in vacuo [21]. The obtained hydrolysates of compounds **1**–**3** and standard amino acids of the *L*- and *D*-configurations (0.2 mg each) were dissolved in 0.1 mL of distilled water, then 0.4 mL of 1M NaHCO_3_ and 0.2 mL of a 1% solution of Marfey’s reagent in acetone were added. The reaction mixtures were kept at 37 °C for 75 min and 0.05 mL of 1M HCl was added. Then, obtained *L*-FDDA derivatives were analyzed by HPLC-UV in gradient of MeCN-H_2_O from 25:75 to 65:35 over 40 min at 20 °C using YMC C-18 Pro column.

### 3.6. The Effect of Compounds ***1**–**3*** on Sortase A Enzymatic Activity

The enzymatic activity of sortase A from *Staphylococcus aureus* was determined using SensoLyte 520 Sortase A Activity Assay Kit * Fluorimetric * (AnaSpec AS-72229, AnaSpec, San Jose, CA, USA) in accordance with the manufacturer’s instructions. DMSO at a concentration of 0.8% was used as a control. Fluorescence was measured with the plate reader PHERAStar FS (BMG Labtech, Offenburg, Germany) for 60 min with a time interval of 5 min. The data were processed by MARS Data Analysis v. 3.01R2 (BMG Labtech, Offen-burg, Germany). The results were presented as relative fluorescent units (RFUs) and percentage of the control data [28].

### 3.7. Cell Lines and Culture Conditions

The human prostate cancer PC-3, human breast cancer MCF-7, and human colorectal cancer DLD-1 cells were purchased from ATCC. The rat cardiomyocytes H9c2 line cells were kindly provided by Prof. Dr. Gunhild von Amsberg from Martini-Klinik Prostate Cancer Center, University Hospital Hamburg-Eppendorf, Hamburg, Germany. The cells were cultured in DMEM medium (Biolot, St. Petersburg, Russia) containing 10% fetal bovine serum (Biolot, St. Petersburg, Russia) and 1% penicillin/streptomycin (Invitrogen, Carlsbad, CA, USA) at 37 °C in a humidified atmosphere with 5% (*v*/*v*) CO_2_. Initially, cells were incubated in cultural flasks until sub-confluent (~80%). For testing, the cells were seeded at concentrations of 5 × 10^3^ cells/well (MCF-7, DLD-1, and PC-3 cells) or 1 × 10^3^ cells/well (H9c2 cells), and experiments were started after 24 h (MCF-7, DLD-1, and PC-3 cells) or 48 h (H9c2 cells).

### 3.8. In Vitro MTT-Based Cytotoxicity Assay

The in vitro cytotoxicity of individual substances was determined by the MTT (3-(4,5-dimethylthiazol-2-yl)-2,5-diphenyltetrazolium bromide) method, according to the manufacturer’s instructions (Sigma-Aldrich, St. Louis, MO, USA).

Investigated compounds were dissolved in DMSO at a concentration of 10 mM. This solution was used to obtain the required concentration of compounds in the cell suspension, so that the concentration of DMSO in the cell suspension would not exceed 1%.

The cells were treated with the investigated compounds for 24 h, and MTT reagent was added to each well of the plate. The vehicle with DMSO at the concentration of 1% was used as a control. The absorbance of formed formazan was measured at λ = 570 nm, using a Multiskan FC microplate photometer (Thermo Scientific, Waltham, MA, USA) and expressed in optical units (o.u.). The results were calculated as % of viable cells to vehicle data.

### 3.9. Statistical Data Evaluation

All results were given as a mean ± standard error of the mean (SEM). General statistical analysis was performed using Student’s *t*-test, employed with the aid of SigmaPlot 14.0 (Systat Software Inc., San Jose, CA, USA). Differences were considered statistically significant at *p* < 0.05.

## Figures and Tables

**Table 1:** ^1^H and ^13^C NMR spectroscopic data (δ in ppm, CDCl_3_) for **1**.

	Pos.	δ_C_, Mult	δ_H_ (*J* in Hz)	HMBC	COSY	ROESY
Ile	1	(NH)	5.57, brs	2, 7, 24, 32	2	2, 24
	2	59.4, CH	2.54, d (2.7)	3, 4, 5, 7	3	5, 6
	3	38.2, CH	1.97, m	4, 5, 7	2, 4, 5	1, 4, 6
	4	15.7, CH_3_	0.89, d (7.2)	2, 3, 5	3	1, 3
	5	24.1, CH_2_	1.24, m 1.40, m	2, 3, 4, 6, 10 2, 3, 4, 6		2
	6	12.1, CH_3_	0.85, t (7.4)	3, 5	5a, 5b	2, 3
	7	170.1, C				
Pro	9	174.7, C				
	10	61.7, CH	5.10, dd (8.9, 4.1)	9, 11, 12, 13 *	11	2, 11
	11	29.7, CH_2_	2.14, m 2.50, m	9, 10, 12, 13 9, 10, 12, 13	10, 12 10, 12	13
	12	24.4, CH_2_	2.09, m 2.17, m	10, 11, 13 10, 11, 13	11, 13 11, 13	13
	13	47.6, CH_2_	3.79, dt (9.2, 7.1) 3.90, m	11, 12 11, 12	12 12	11, 12, 16, 17
CA	15	164.6, C				
	16	117.9, CH	6.73, d (15.5)	15, 17, 18	17	13, 22, 23
	17	142.8, CH	7.66, d (15.6)	16, 18, 19, 23	16	13
	18	135.1, C				
	19	127.9, CH	7.51	17, 21, 23	20	
	20	128.8, CH	7.36, m	18, 22	19, 21	
	21	129.7, CH	7.35, m	19, 23	20, 22	
	22	128.8, CH	7.36, m	18, 20	21, 23	
	23	127.9, CH	7.51	17, 19, 21	22	
Phe	24	58.2, CH	5.22, t (4.5)	7, 9 *, 25, 26, 32	25	25
	25	38.5, CH_2_	3.28, dd (14.0, 4.2) 3.33, dd (14.0, 4.9)	24, 26, 27, 31, 32 24, 26, 27, 31, 32	24 24	11, 24, 26, 27
	26	135.0, C				
	27	130.5, CH	7.10, m	25, 29, 31	28	25
	28	128.7, CH	7.29, m	26, 30	27, 29	25
	29	127.8, CH	7.30, m	27, 31	28, 30	
	30	128.7, CH	7.29, m	26, 28	29, 31	
	31	130.5, CH	7.10, m	25, 27, 29	30	
	32	168.2, C				

*—weak interaction.

**Table 2:** ^1^H and ^13^C NMR spectroscopic data (δ in ppm, CDCl_3_) for **2**.

	Pos.	δ_C_, Mult	δ_H_ (*J* in Hz)	HMBC	COSY	ROESY
Leu	1	(NH)	5.62, brs	2, 7, 24, 32	2	2, 24
	2	52.6, CH	2.48, dd (8.8, 3.8)	3, 4, 5, 7	3	5, 6
	3	40.7, CH_2_	1.67, m 1.52, m	2, 3, 4, 5, 6, 7, 32	2, 4, 5	1, 4, 6
	4	24.4, CH	2.07, m	2, 3, 5, 6		2
	5	23.1, CH_3_	0.88, d (6.2)	2, 3, 6	3	1, 3
	6	20.7, CH_3_	0.70, d (6.3)	3, 5	5a, 5b	2, 3
	7	170.8, C				
Pro	9	175.0, C				
	10	61.8, CH	5.17, dd (8.6, 3.6)	9, 13 *	11	2, 11
	11	29.7, CH_2_	2.12, m 2.48, dd (8.8, 3.8)	9, 10, 12, 13 9, 10, 12, 13	10, 12 10, 12	13
	12	29.6, CH_2_	2.11, m 1.26, m	10, 11, 13 10, 11, 13	11, 13 11, 13	13
	13	47.6, CH_2_	3.93, m 3.79, dd (16.3, 7.3)	11, 12 11, 12	12 12	11, 12, 16, 17
CA	15	164.6, C				
	16	117.8, CH	6.73, d (15.4)	15, 17, 18	17	13, 22, 23
	17	142.9, CH	7.66, d (15.5)	16, 18, 19, 23	16	13
	18	135.1, C				
	19	127.9, CH	7.52, d (2.3)	17, 21, 23	20	
	20	128.7, CH	7.29, m	18, 22	19, 21	
	21	129.8, CH	7.36, m	19, 23	20, 22	
	22	128.7, CH	7.29, m	18, 20	21, 23	
	23	127.9, CH	7.52, d (2.3)	17, 19, 21	22	
Phe	24	58.6, CH	5.23, t (4.5)	7, 9, 25, 26, 32	25	25
	25	38.4, CH_2_	3.28, dd (14.0, 4.9) 3.35, dd (14.0, 5.0)	24, 26, 27, 31, 32 24, 26, 27, 31, 32	24 24	11, 24, 26, 27
	26	135.2, C				
	27	130.4, CH	7.13, d (1.8)	25, 29, 31	28	25
	28	128.8, CH	7.29, m	26, 30	27, 29	25
	29	127.7, CH	7.36, m	27, 31	28, 30	
	30	128.8, CH	7.29, m	26, 28	29, 31	
	31	130.4, CH	7.13, d (1.8)	25, 27, 29	30	
	32	168.1, C				

*—weak interaction.

**Table 3:** ^1^H and ^13^C NMR spectroscopic data (δ in ppm, CDCl_3_) for **3**.

	Pos.	δ_C_, Mult	δ_H_ (*J* in Hz)	HMBC	COSY	ROESY
Val	1	(NH)	5.67, brs	2, 8, 23, 31	2	2, 24
	2	58.0, CH	2.60, d (2.7)	3, 4, 5, 7	3	5
	3	31.7, CH	2.31, m	2, 3, 4, 5, 7, 31	2, 4, 5	1, 4
	4	15.8, CH_3_	0.93, d (6.8)	2, 3, 5	3	1, 3
	5	18.9, CH_3_	0.90, d (7.1)	2, 3, 4		2
	7	170.2, C				
Pro	9	174.6, C				
	10	61.6, CH	5.11, dd (8.6, 3.7)	9, 11, 12	11	2, 11
	11	29.7, CH_2_	2.14, m 2.48, m	9, 10, 12 9, 12	10, 12 10, 12	13
	12	24.4, CH_2_	2.18, m 2.08, m	10, 13 10, 13		12b 12a, 12b
	13	47.6, CH_2_	3.79, m 3.94, m	11, 12 11, 12	10, 11b, 12a, 12b 10, 11b, 12a, 12b	15, 11b 15, 11a
	15	164.6, C				
CA	16	117.8, CH	6.72, d (15.5)	15, 17, 18, 19/23	17	13a, 13b, 19/23, 20/22
	17	142.9, CH	7.66, d (15.6)	15, 16, 18, 19/23	16	13a, 13b
	18	135.1, C				
	19	127.9, CH	7.50, d (6.9)	21, 23	20	16
	20	128.8, CH	7.35, brd (1.4)	17, 21	19, 21	16
	21	129.7, CH	7.34, m	19, 23	20, 22	
	22	128.8, CH	7.35, brd (1.4)	18, 20	21, 23	16
	23	127.9, CH	7.51, d (7.8)	17, 19, 21		16
Phe	24	59.8, CH	5.22, t (4.5)	7, 9, 25, 26, 32		
	25	38.6, CH_2_	3.28, dd (14.0, 5.0) 3.34, dd (14.0, 4.0)	25, 27/31, 32 25, 27/31, 32	29 29	27/31, 28 27/31, 28
	26	134.9, C				
	27	130.6, CH	7.10, d (1.7)	25, 27, 29		10, 24
	28	128.7, CH	7.29, m	26, 29, 31		
	29	127.9, CH	7.29, m	27, 31		
	30	128.7, CH	7.29, m	26, 27, 29		
	31	130.6, CH	7.10, d (1.7)	25, 27, 29		10, 24
	32	168.2, C				

**Table 4:** Cytotoxic activity of asterripeptides A–C (**1**–**3**).

Compound	IC_50_, µM			
	MCF-7	DLD-1	PC-3	H9c2
**1**	96.8 ± 7.0	87.7 ± 5.3	64.6 ± 2.4	76.7 ± 5.2
**2**	>100	>100	75.5 ± 1.9	104.1 ± 3.3
**3**	96.6 ± 1.5	84.9 ± 7.4	58.3 ± 3.2	87.6 ± 4.5

All experiments were carried out in independent three experiments. Data are presented as a mean ± standard mean error.

## Data Availability

Not applicable.

## Supplementary Materials

The following supporting information can be downloaded at: https://www.mdpi.com/article/10.3390/md20010077/s1, Figures S1–S7, S9–S15 and S17–S23: NMR spectra of compounds **1**–**3**; Figures S8, S16 and S24: MS data for compounds **1**–**3**; Figures S25–S36: HPLC profiles of FDAA derivatives for compounds **1**–**3**.
