# Supplementary material for: New Tripeptide Derivatives Asperripeptides A–C from Vietnamese Mangrove-Derived Fungus Aspergillus terreus LM.5.2
Source: Mar Drugs. 2022 Jan 17;20(1):77. doi: 10.3390/md20010077 (PMC8780021; doi:10.3390/md20010077)

SUPPLEMENTARY MATERIAL

Tripeptide Derivatives from Vietnamese Mangrove-Derived Fungus *Aspergillus terreus* LM.5.2

Elena V. Girich[1], Anton B. Rasin[1], Roman S. Popov[1], Ekaterina A. Yurchenko[1], Ekaterina A. Chingizova[1], Phan Thi Hoai Trinh[2], Ngo Thi Duy Ngoc[2], Mikhail V. Pivkin[1], Olesya I. Zhuravleva[3] and Anton N. Yurchenko[1,*]

[1] G.B. Elyakov Pacific Institute of Bioorganic Chemistry, Far Eastern Branch of the Russian Academy of Sciences, Prospect 100-letiya Vladivostoka, 159, Vladivostok 690022, Russia; ev.ivanets@yandex.ru (E.V.G.), yurchant@ya.ru (A.N.Y.); abrus__54@mail.ru (A.B.R.); prs_90@mail.ru (R.S.P.); dminae@mail.ru (E.A.Y.), maryyas@mail.ru (E.A.C.), oid27@mail.ru (M.V.P.)

[2] Department of marine biotechnology, Nhatrang Institute of Technology Research and Application, Vietnam Academy of Science and Technology, Nha Trang, Vietnam; phanhoaitrinh84@gmail.com (P.T.H.T.); ngocnitra@gmail.com (N.T.D.N.)

[3] School of Natural Sciences, Far Eastern Federal University, Vladivostok, Russian Federation; zhuravleva.oi@dvfu.ru (O.I.Z.)

* Correspondence: yurchant@ya.ru; Tel.: +7-423-231-1168

Received: date; Accepted: date; Published: date

Abstract: Three new tripeptide-derivatives containing in their structures a rare cinnamic acid residue asterripeptides A-C (1-3) were isolated from Vietnamese mangrove-derived fungus *Aspergillus terreus* LM 5.2. Structures of isolated compounds were determined by NMR and ESIMS techniques combination. Absolute configurations of all stereocenters were determined using the Murfey's method via determining of amino acids configuration. Cytotoxicity against several cancer cell lines and inhibition ability of sortase A from Staphylococcus aureus of asterripeptides A-C were investigated.

Keywords: marine-derived fungi, mangrove-derived fungi, secondary metabolites, diketopiperazines, tripeptide derivatives, cinnamic acid, sortase A, cytotoxicity

Content

Figure S1. ^{1}H NMR spectrum of asterripeptide A (1) 4
Figure S2. ^{13}C NMR spectrum of asterripeptide A (1) 6
Figure S3. DEPT-135 spectrum of asterripeptide A (1) 7
Figure S4. ^{1}H-^{1}H COSY spectrum of asterripeptide A (1) 8
Figure S5. HMBC spectrum of asterripeptide A (1) 9
Figure S6. HSQC spectrum of asterripeptide A (1) 10
Figure S7. ROESY spectrum of asterripeptide A (1) 11
Figure S8. MS and MS/MS data of asterripeptide A (1) 12
Figure S9. ^{1}H NMR data of asterripeptide B (2) 13
Figure S10. ^{13}C NMR spectrum of asterripeptide B (2) 15
Figure S11. DEPT NMR spectrum of asterripeptide B (2) 16
Figure S12. HSQC spectrum of asterripeptide B (2) 17
Figure S13. HMBC spectrum of asterripeptide B (2) 18
Figure S14. ^{1}H-^{1}H COSY spectrum of asterripeptide B (2) 19
Figure S15. NOESY spectrum of asterripeptide B (2) 20
Figure S16. MS and MS/MS data of asterripeptide B (2) 21
Figure S17. ^{1}H NMR spectrum of asterripeptide C (3) 22
Figure S18. ^{13}C NMR spectrum of asterripeptide C (3) 24
Figure S19. DEPT NMR spectrum of asterripeptide C (3) 25
Figure S20. HSQC NMR spectrum of asterripeptide C (3) 26
Figure S21. HMBC NMR spectrum of asterripeptide C (3) 27
Figure S22. ^{1}H-^{1}H COSY NMR spectrum of asterripeptide C (3) 28
Figure S23. ROESY NMR spectrum of asterripeptide C (3) 29
Figure S24. MS and MS/MS data of asterripeptide C (3) 30
Figure S25. HPLC profile of *L*-FDAA-derivatives of asterripeptide A (1) hydrolysis products (HP) 31
Figure S26. HPLC profile of *L*-FDAA-derivatives of asterripeptide A (1) HP (a), asterripiptide A HP+*L*-Ile (b) and asterripiptide A HP+*D*,*L*-Ile (c) 32
Figure S27. HPLC profile of *L*-FDAA-derivatives of asterripeptide A (1) HP, asterripiptide A HP+*L*-Phe and asterripiptide A HP+*D*,*L*-Phe 33
Figure S28. HPLC profile of *L*-FDAA-derivatives of asterripeptide A (1) HP, asterripiptide A HP+*D*,*L*-Pro and asterripiptide A HP+*L*-Pro 34
Figure S29. HPLC profile of *L*-FDAA-derivatives of asterripeptide B (2) HP 35

Figure S30. HPLC profiles of *L*-FDAA-derivatives of asterripeptide B (2) HP (a), asterripiptide B HP+*L*-Leu (b) and asterripiptide B HP+*D*,*L*-Leu (c)............................ 36

Figure S31. HPLC profiles of *L*-FDAA-derivatives of asterripeptide B (2) HP (a), asterripiptide B+ *D*,*L*-Pro (b) and asterripiptide B+ *L*-Pro (c) 37

Figure S32. HPLC profiles of *L*-FDAA-derivatives of asterripeptide B (2) HP (a), asterripiptide B HP+ *L*-Phe (b) and asterripiptide B HP+ *D*,*L*-Phe (c).......................... 38

Figure S33. HPLC profile of *L*-FDAA-derivatives of asterripeptide C (3) HP 39

Figure S34. HPLC profiles of *L*-FDAA-derivatives of asterripeptide C (3) HP (a), asterripiptide C+*L*-Val H (b)P and asterripiptide C HP+*D*,*L*-Val (c) 40

Figure S35. HPLC profiles of *L*-FDAA-derivatives of asterripeptide C (3) HP (a), asterripiptide C HP+*L*-Pro (b) and asterripiptide C HP+*D*,*L*-Pro (c)............................. 41

Figure S36. HPLC profiles of *L*-FDAA-derivatives of asterripeptide C (3) HP (a), asterripiptide C HP+ *D*,*L*-Phe (b) and asterripiptide C HP+*L*-Phe (c)........................... 42

Figure S1. ¹H NMR spectrum of asterripeptide A (1)

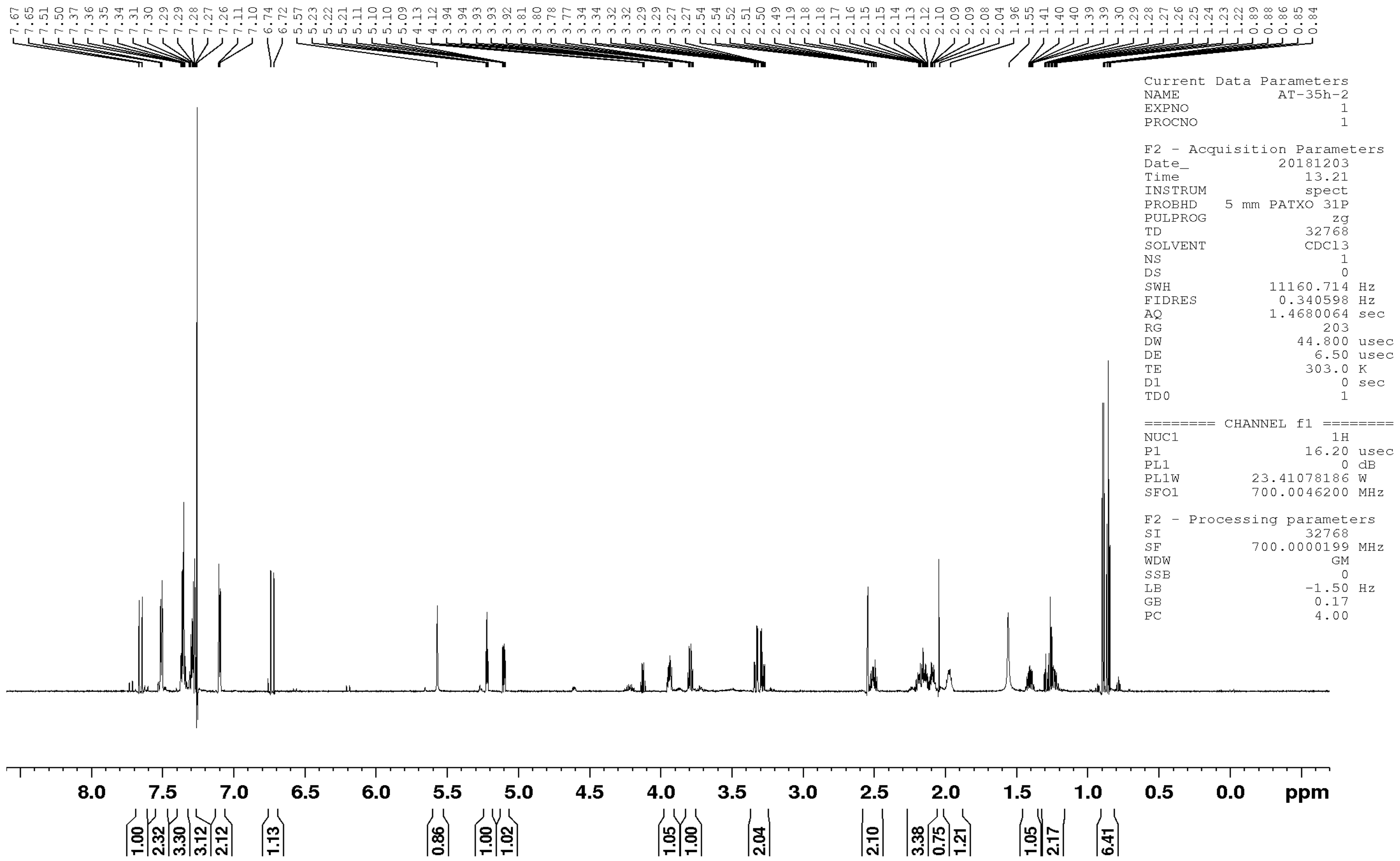

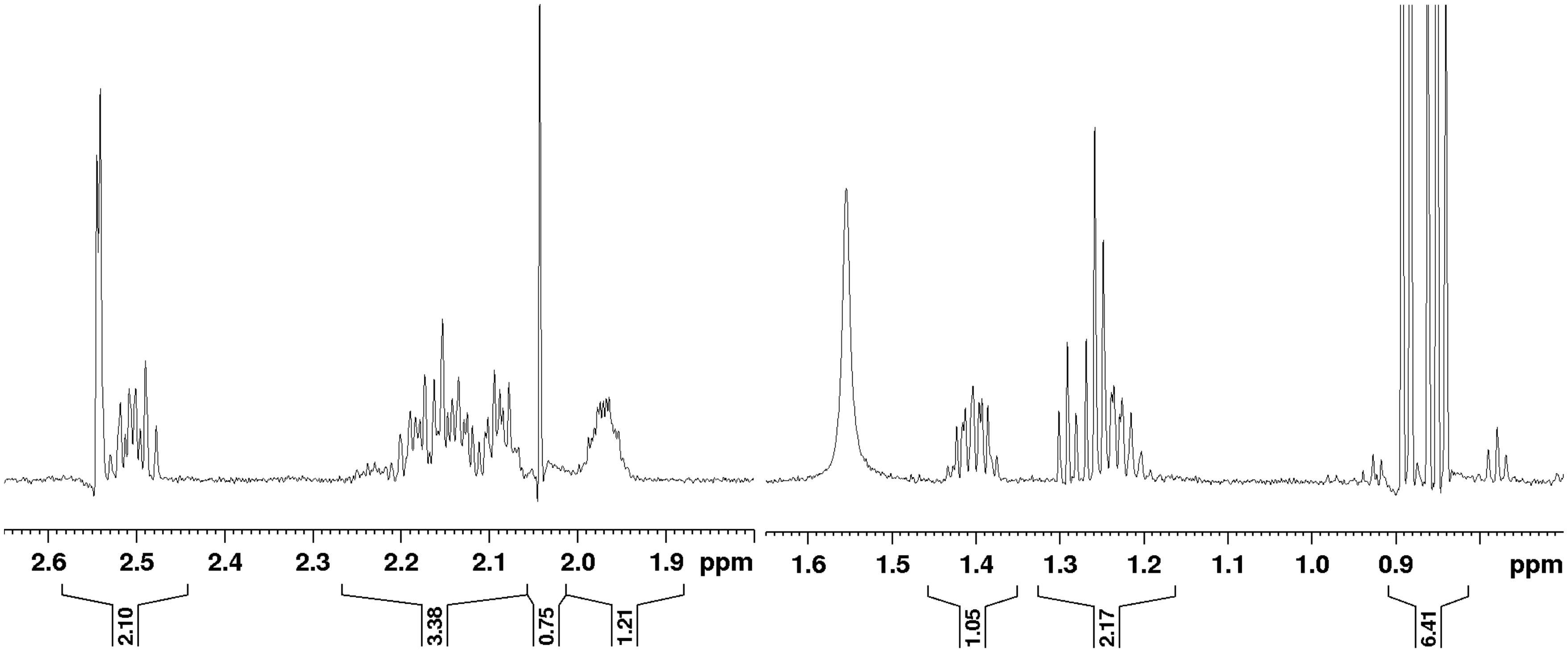
2.6 2.5 2.4 2.3 2.2 2.1 2.0 1.9 ppm
1.6 1.5 1.4 1.3 1.2 1.1 1.0 0.9 ppm
2.10
3.38
0.75
1.21
1.05
2.17
6.41

Figure S2. ^{13}C NMR spectrum of asterripeptide A (1)

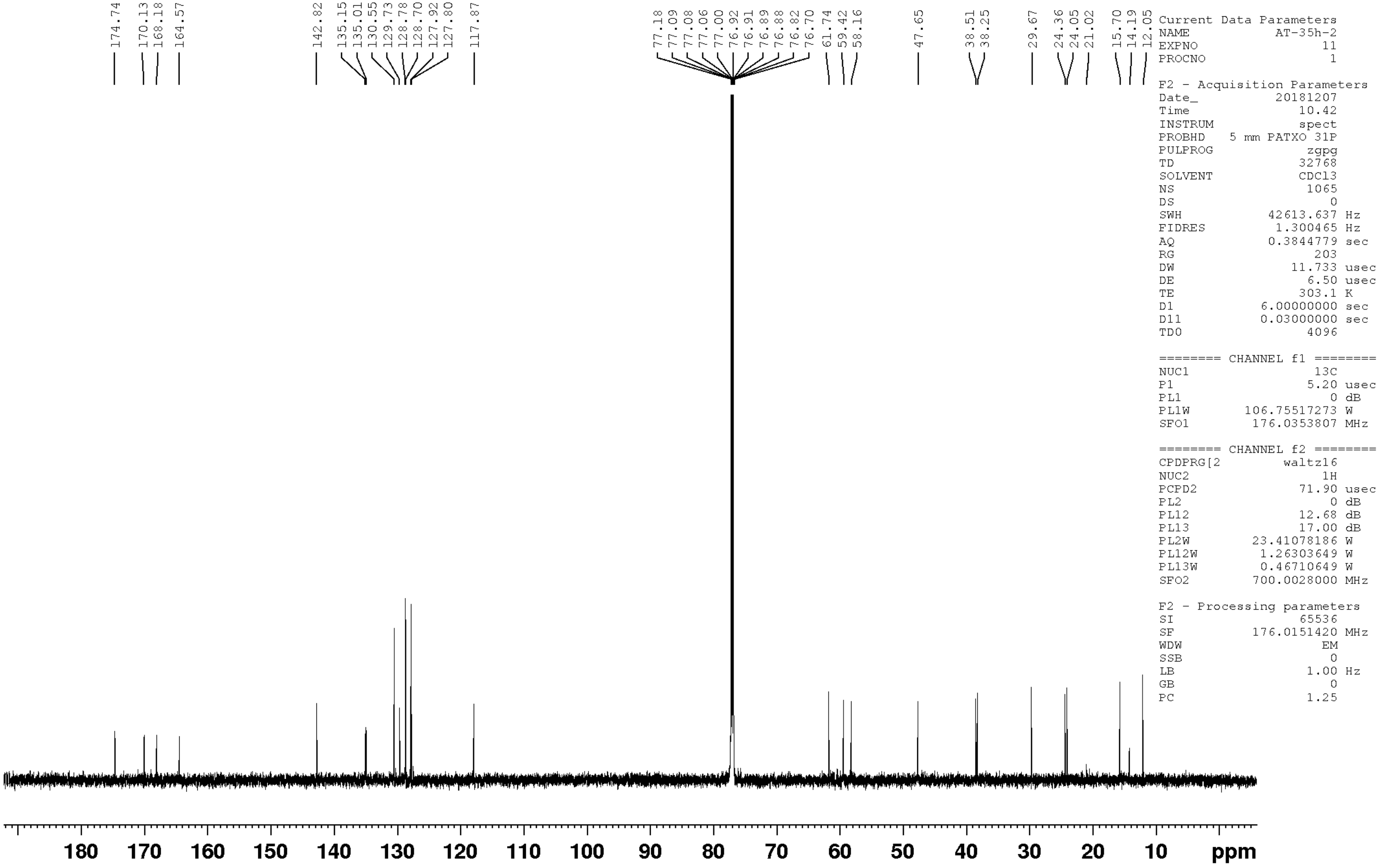

Figure S3. DEPT-135 spectrum of asterripeptide A (1)

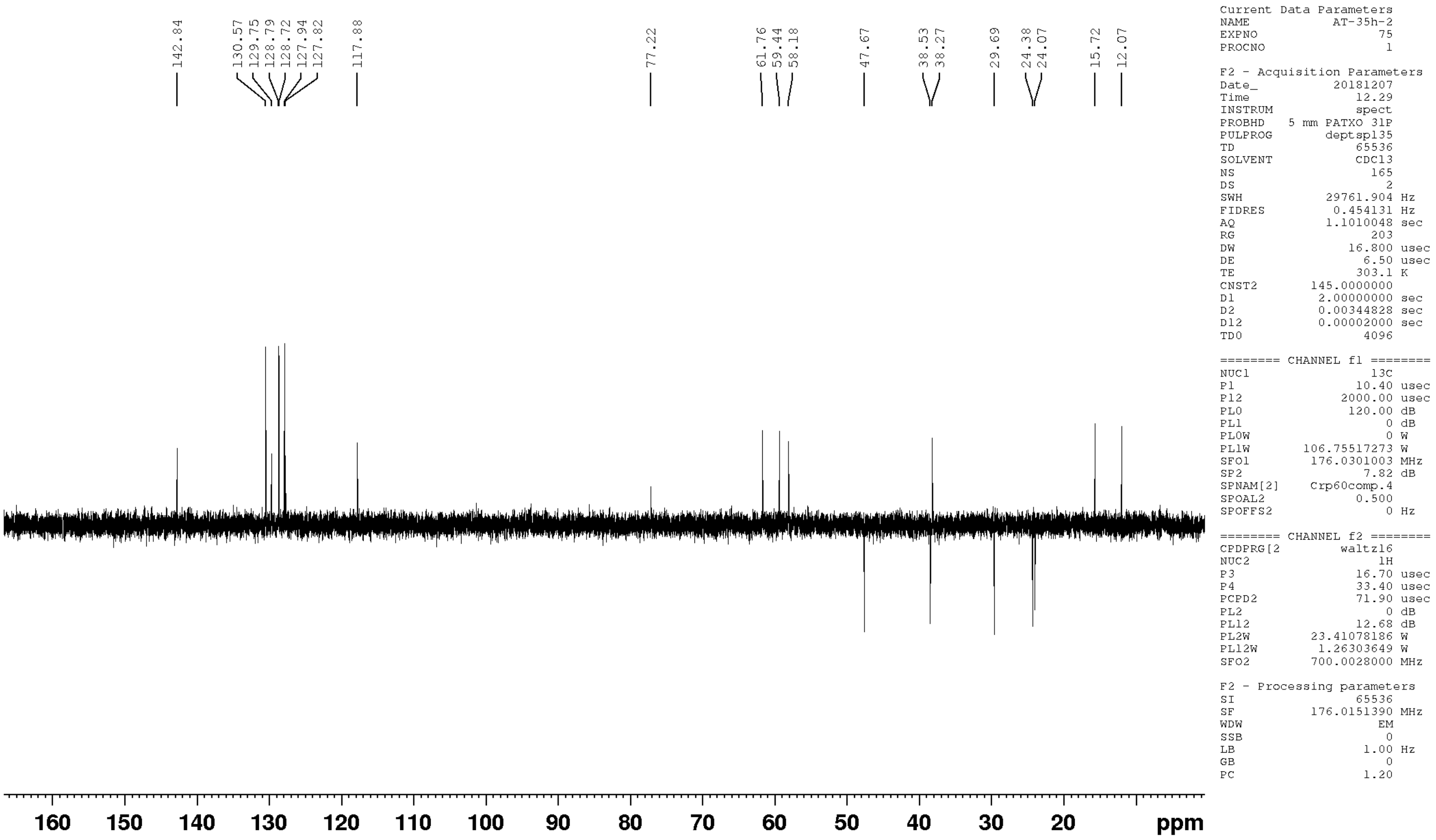

Figure S4. ^{1}H-^{1}H COSY spectrum of asterripeptide A (1)

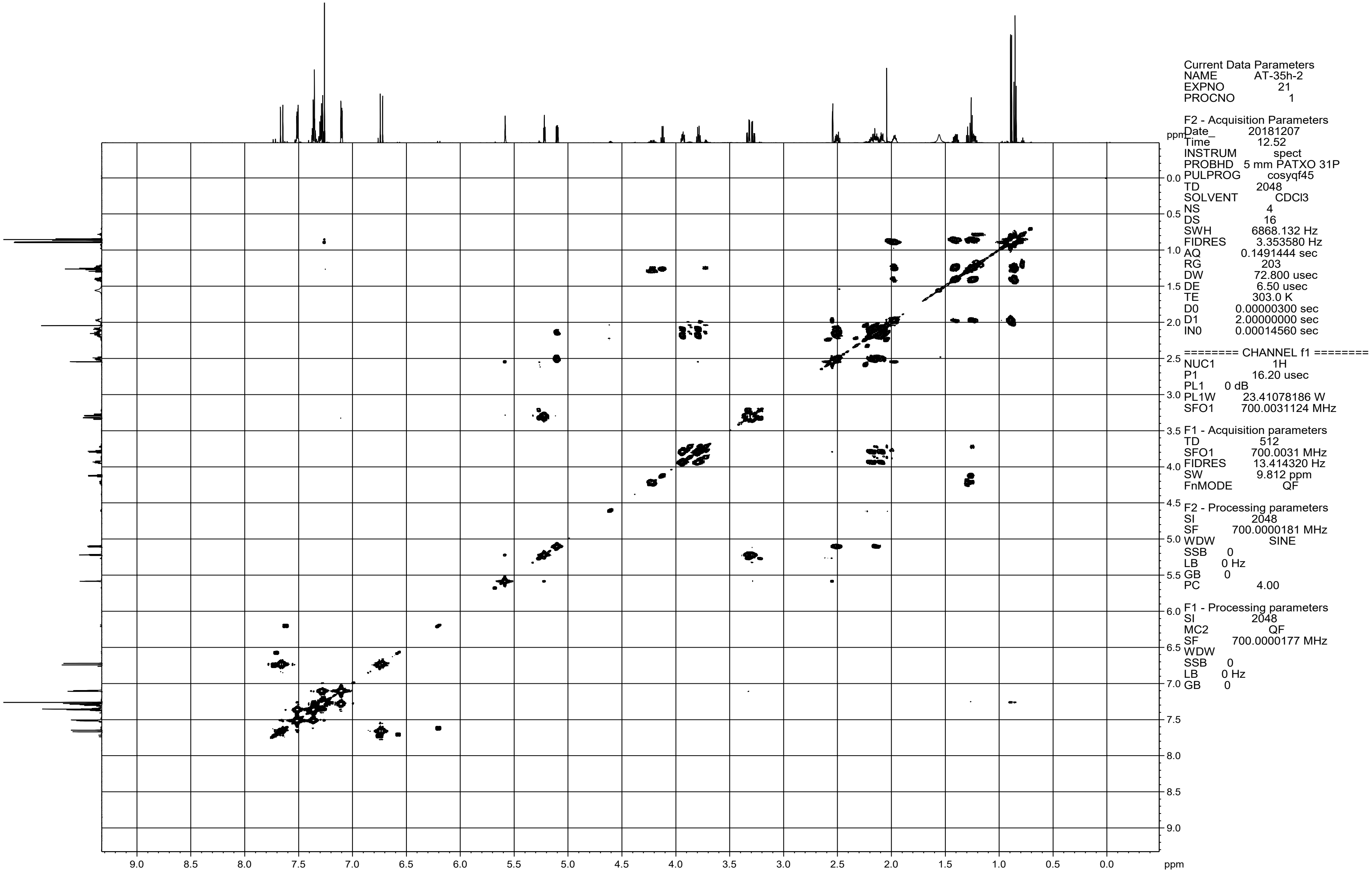

Figure S5. HMBC spectrum of asterripeptide A (1)

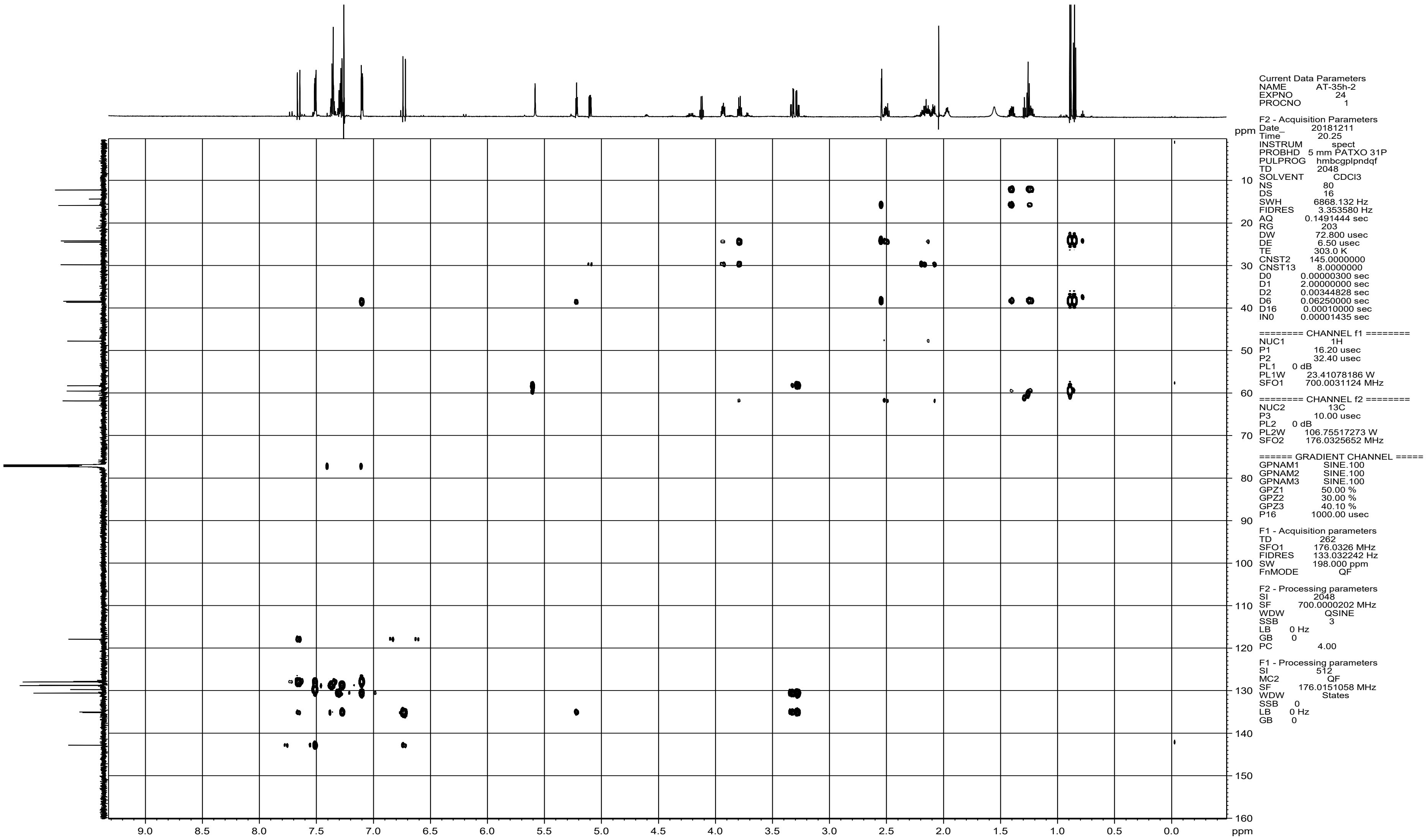

Figure S6. HSQC spectrum of asterripeptide A (1)

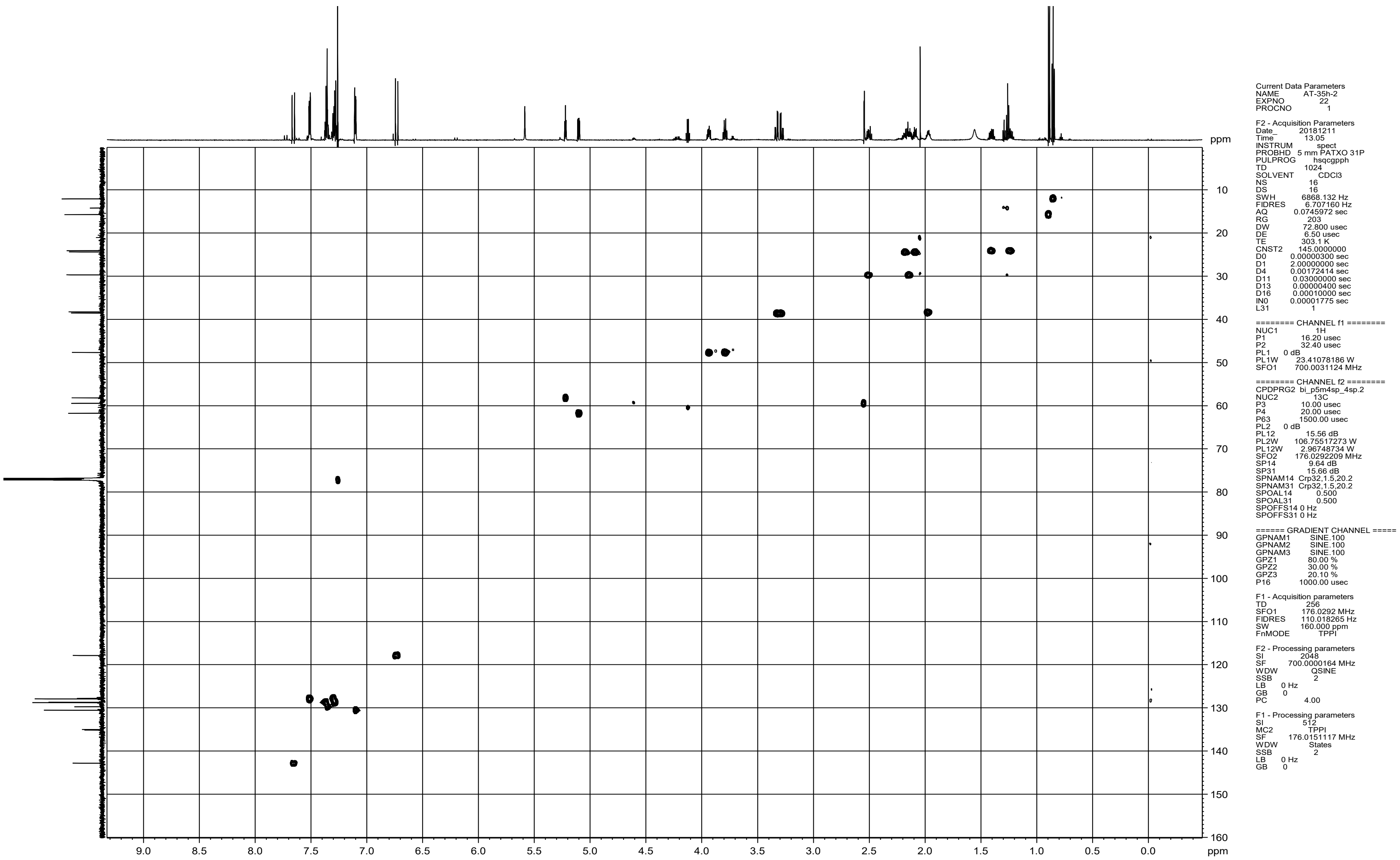

Figure S7. ROESY spectrum of asterripeptide A (1)

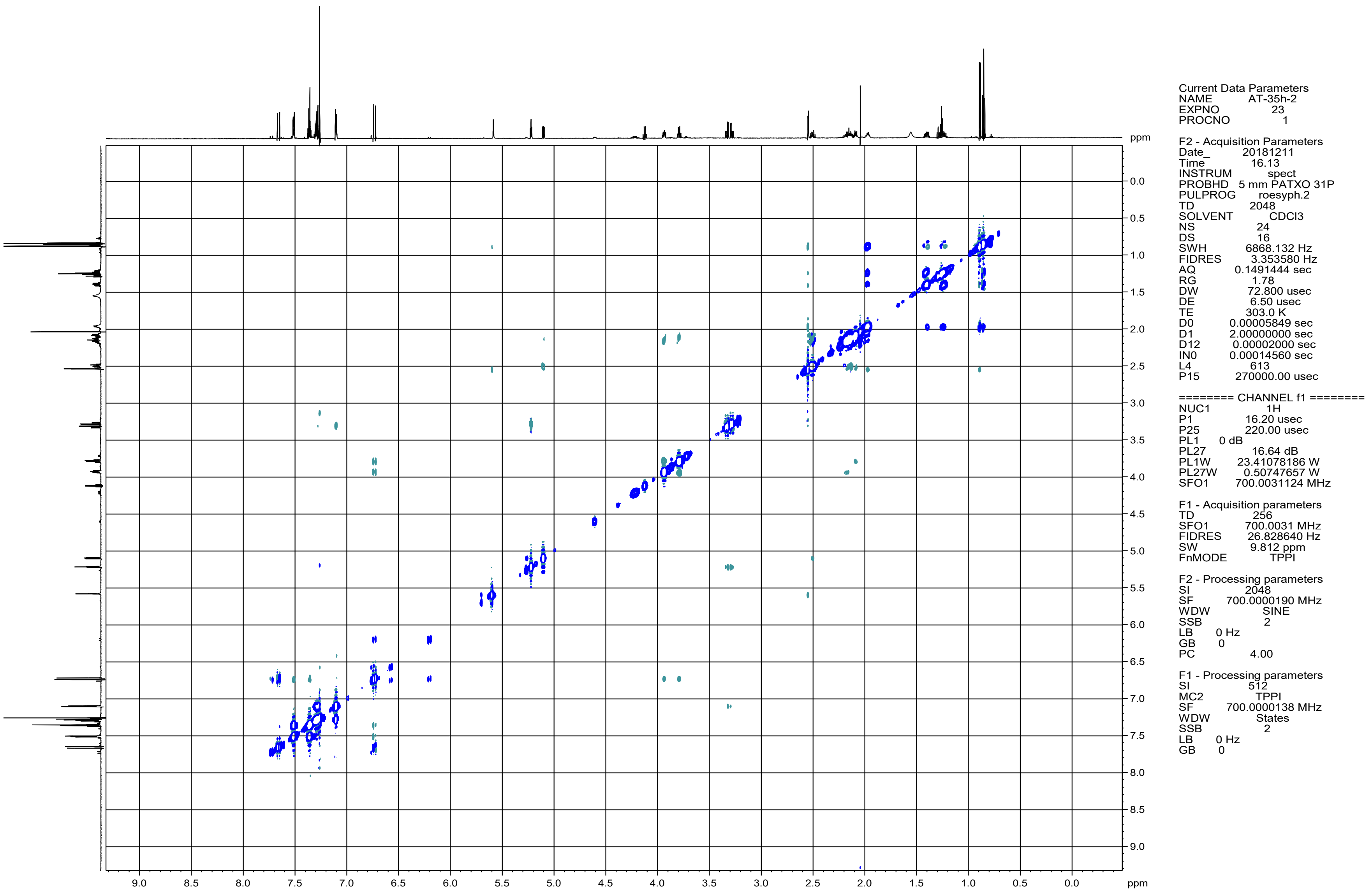

Figure S8. MS and MS/MS data of asterripeptide A (1)

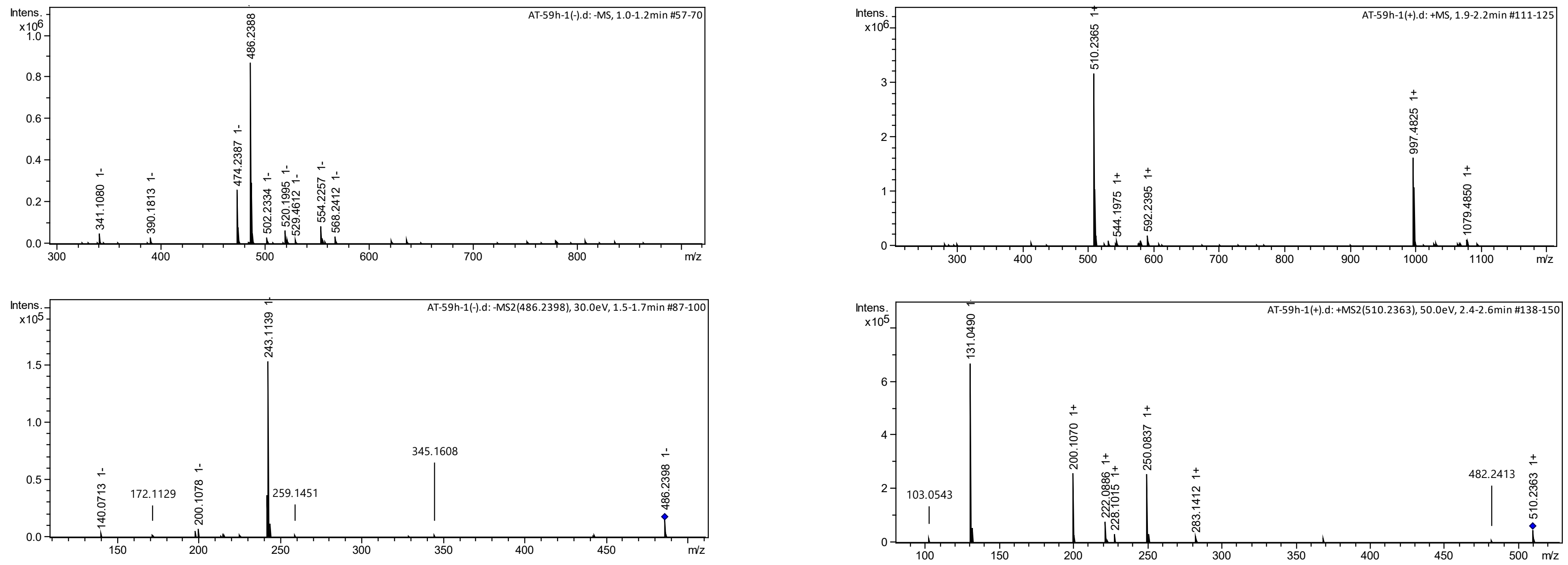

	meas.	calc.	Δ (ppm)
[M-H]$^-$	486,2387	486,2398	2,2
[M+Na]$^+$	510,2365	510,2363	-0,3

Figure S9. ^{1}H NMR data of asterripeptide B (2)

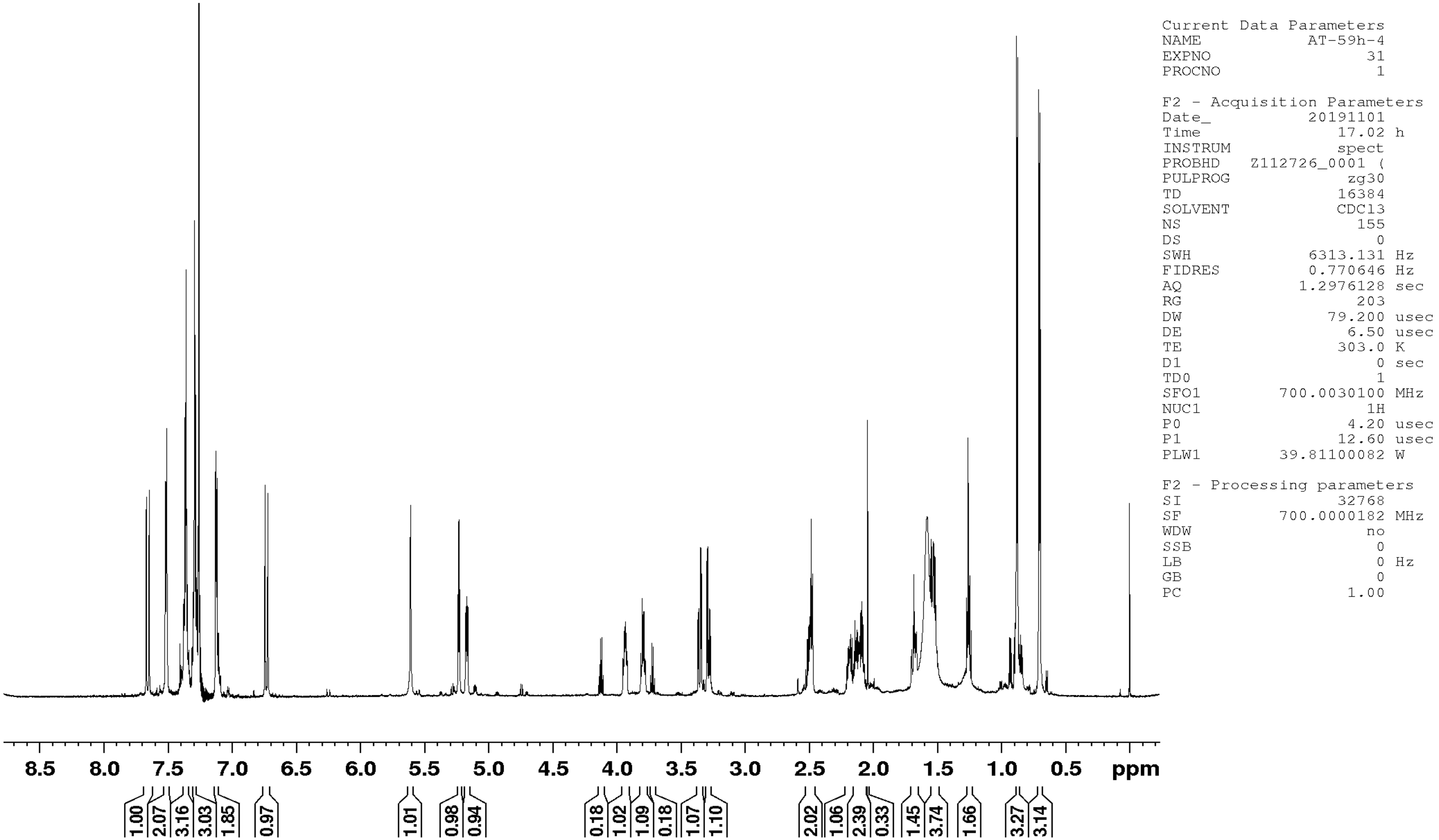

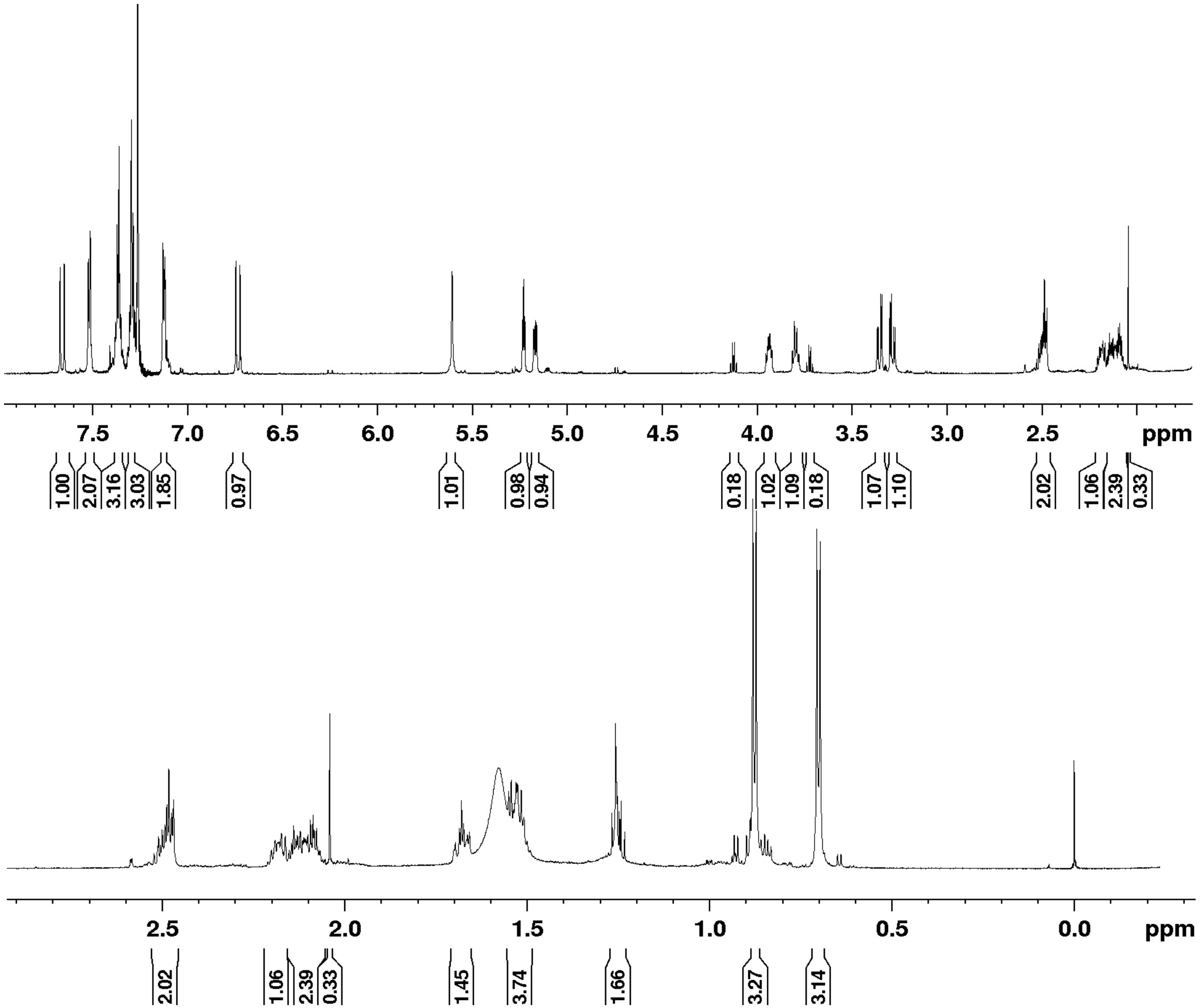


```
Current Data Parameters
NAME              AT-59h-4
EXPNO                   31
PROCNO                   1

F2 - Acquisition Parameters
Date_             20191101
Time                 17.02 h
INSTRUM              spect
PROBHD     Z112726_0001 (
PULPROG               zg30
TD                   16384
SOLVENT              CDCl3
NS                     155
DS                       0
SWH               6313.131 Hz
FIDRES            0.770646 Hz
AQ               1.2976128 sec
RG                     203
DW                  79.200 usec
DE                    6.50 usec
TE                   303.0 K
D1                       0 sec
TD0                      1
SFO1           700.0030100 MHz
NUC1                    1H
P0                    4.20 usec
P1                   12.60 usec
PLW1           39.81100082 W

F2 - Processing parameters
SI                   32768
SF             700.0000182 MHz
WDW                     no
SSB                      0
LB                       0 Hz
GB                       0
PC                    1.00
```

Figure S10. ^{13}C NMR spectrum of asterripeptide B (2)

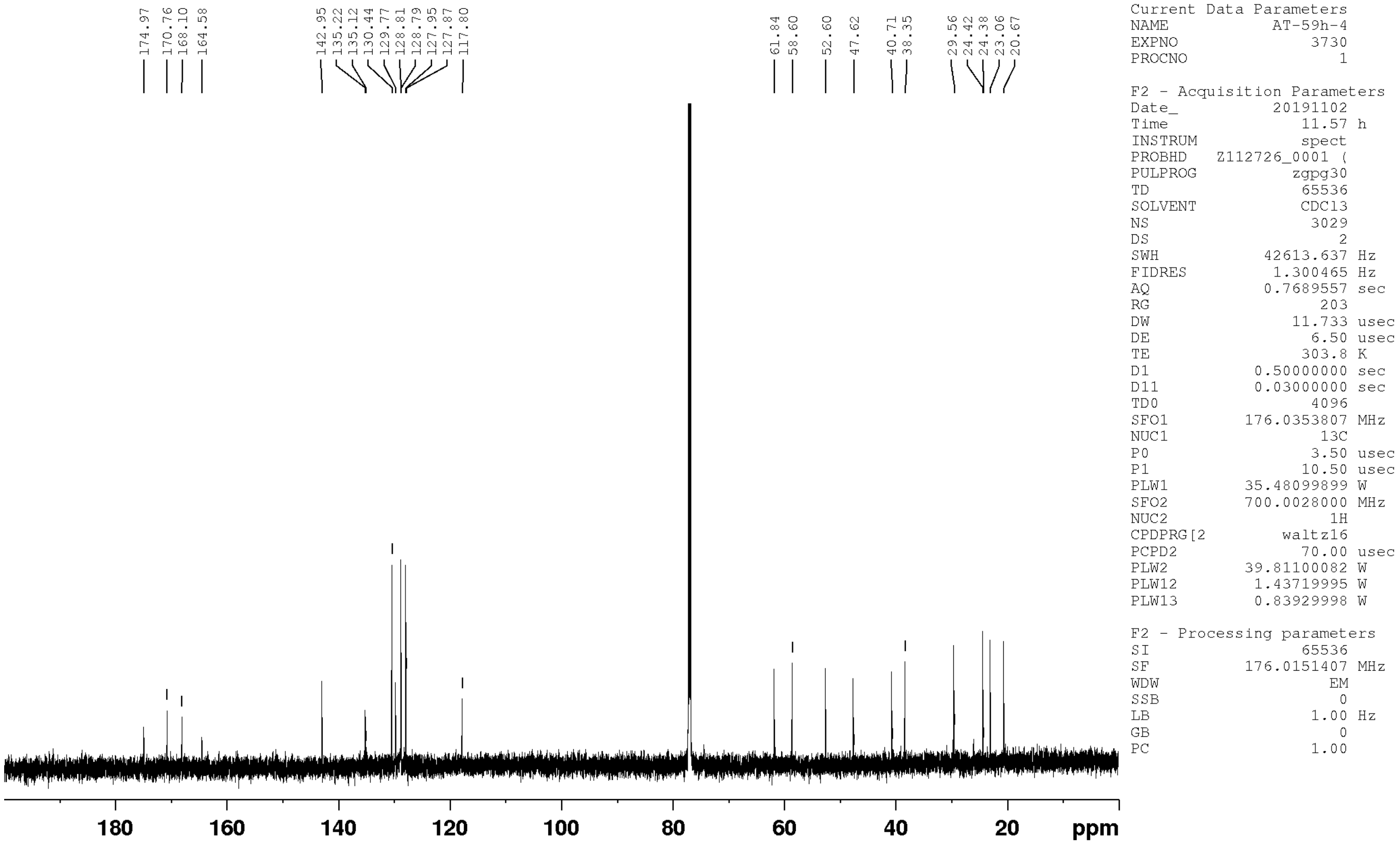

Figure S11. DEPT NMR spectrum of asterripeptide B (2)

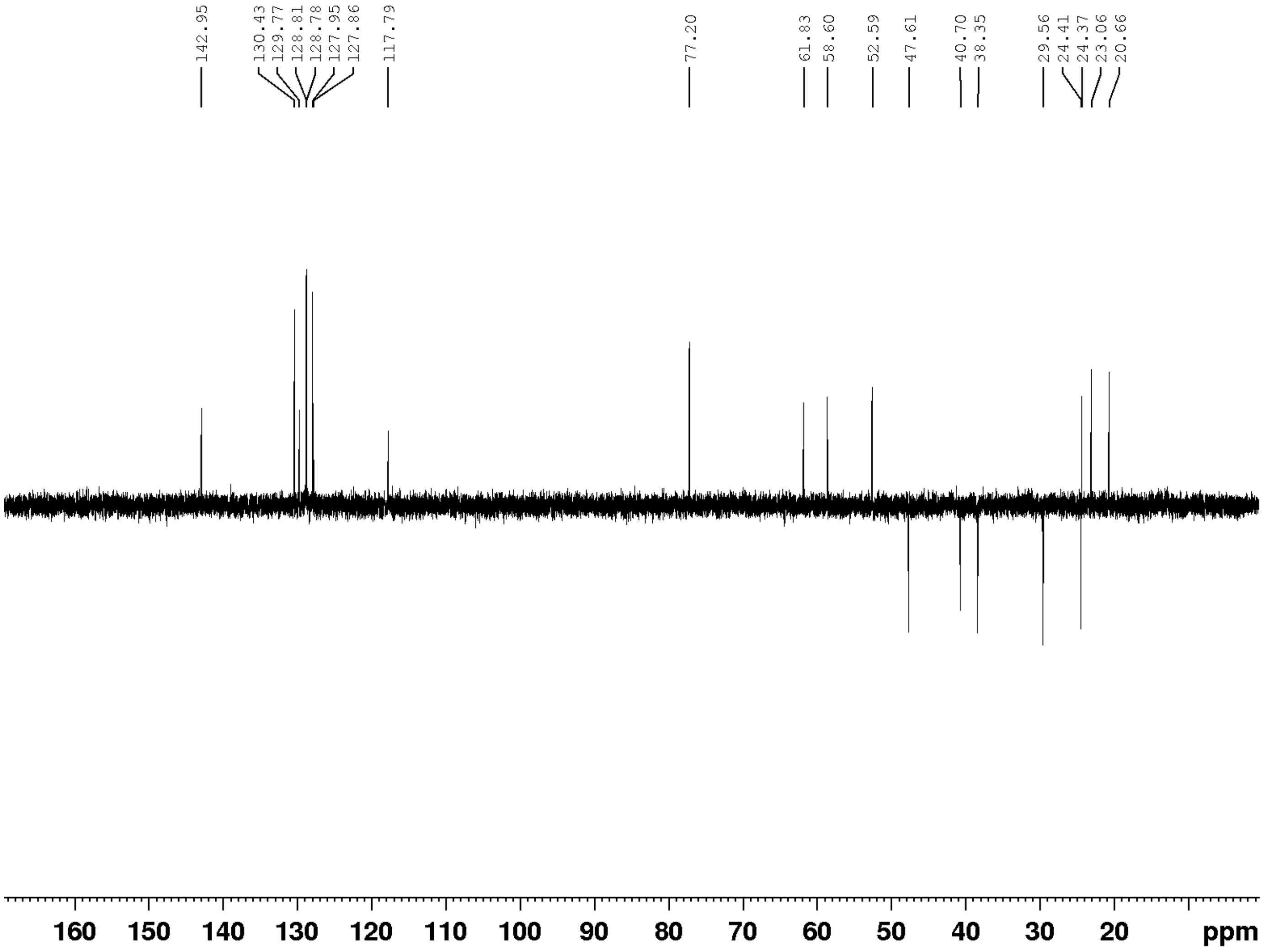

Current Data Parameters
NAME AT-59h-4
EXPNO 3135
PROCNO 1

F2 - Acquisition Parameters
Date_ 20191101
Time 20.50 h
INSTRUM spect
PROBHD Z112726_0001 (
PULPROG deptsp135
TD 65536
SOLVENT CDCl3
NS 1024
DS 2
SWH 29761.904 Hz
FIDRES 0.908261 Hz
AQ 1.1010048 sec
RG 203
DW 16.800 usec
DE 6.50 usec
TE 303.4 K
CNST2 145.0000000
D1 2.00000000 sec
D2 0.00344828 sec
D12 0.00002000 sec
TD0 64
SFO1 176.0301003 MHz
NUC1 13C
P1 10.50 usec
P13 2000.00 usec
PLW0 0 W
PLW1 35.48099899 W
SPNAM[5] Crp80comp.4
SPOAL5 0.500
SPOFFS5 0 Hz
SPW5 7.96899986 W
SFO2 700.0028000 MHz
NUC2 1H
CPDPRG[2 waltz16
P3 13.30 usec
P4 26.60 usec
PCPD2 70.00 usec
PLW2 39.81100082 W
PLW12 1.43719995 W

F2 - Processing parameters
SI 65536
SF 176.0151418 MHz
WDW EM
SSB 0
LB 1.00 Hz
GB 0
PC 1.40

Figure S12. HSQC spectrum of asterripeptide B (2)

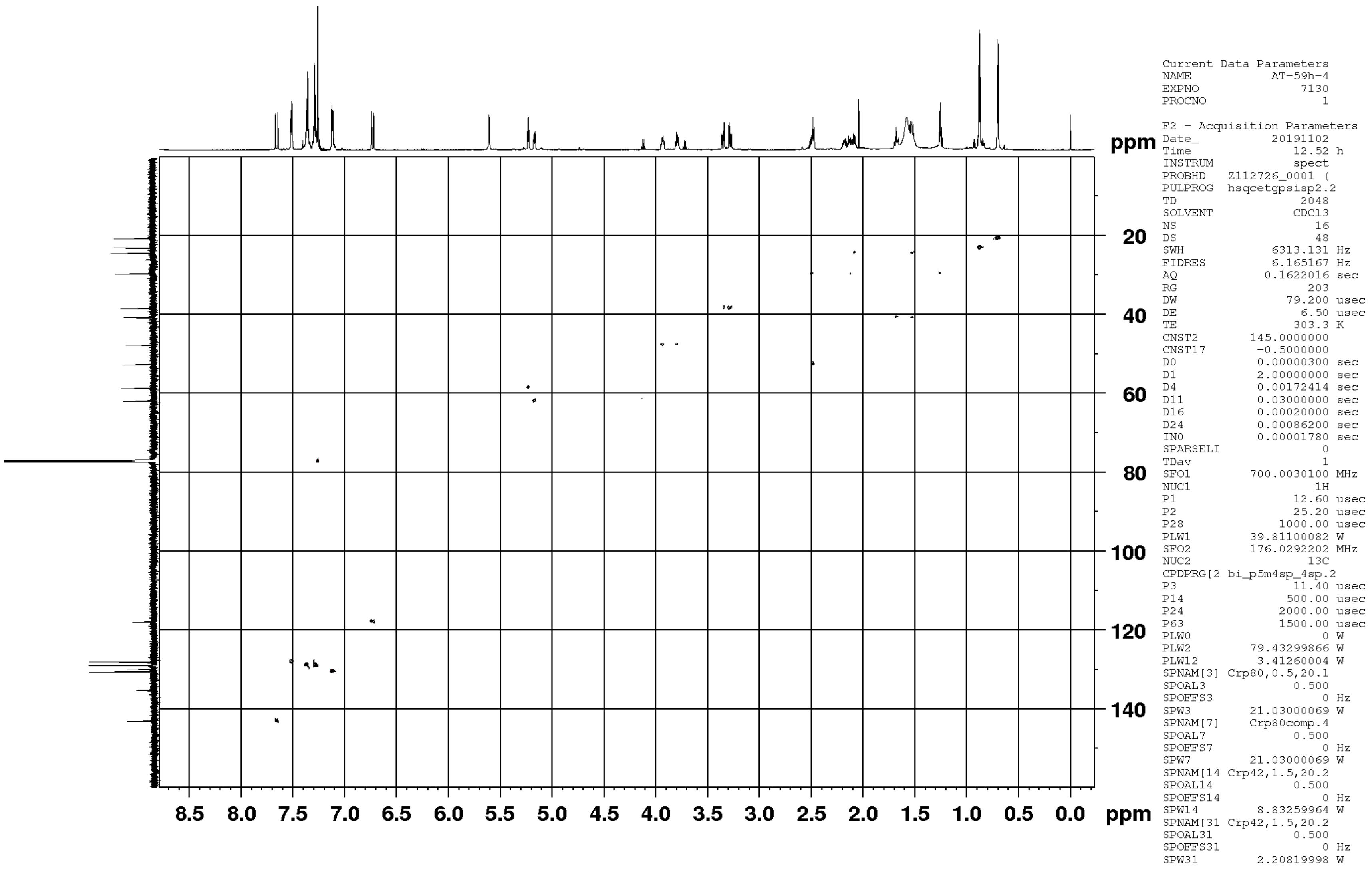

Figure S13. HMBC spectrum of asterripeptide B (2)

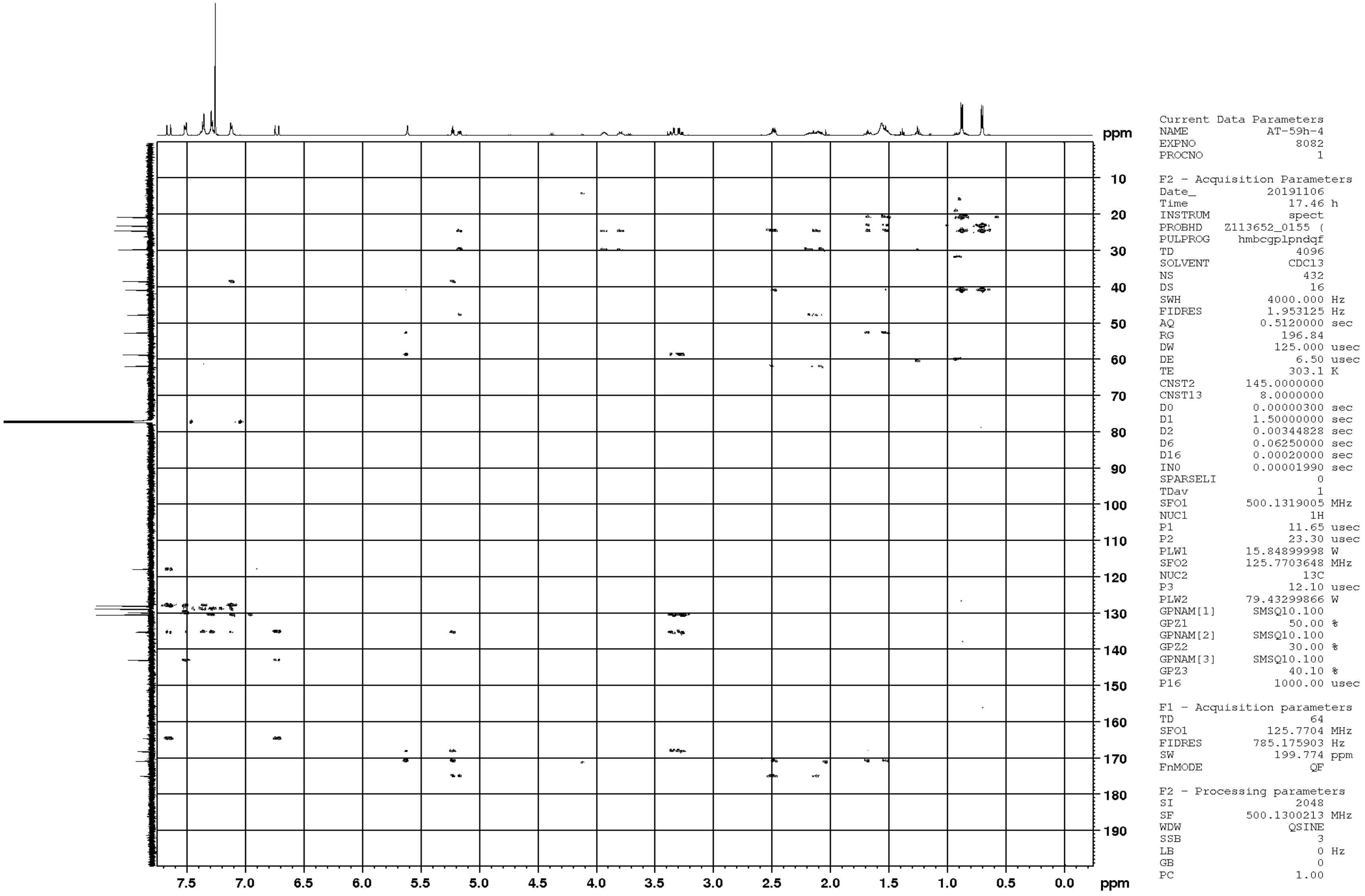

Figure S14. ¹H-¹H COSY spectrum of asterripeptide B (2)

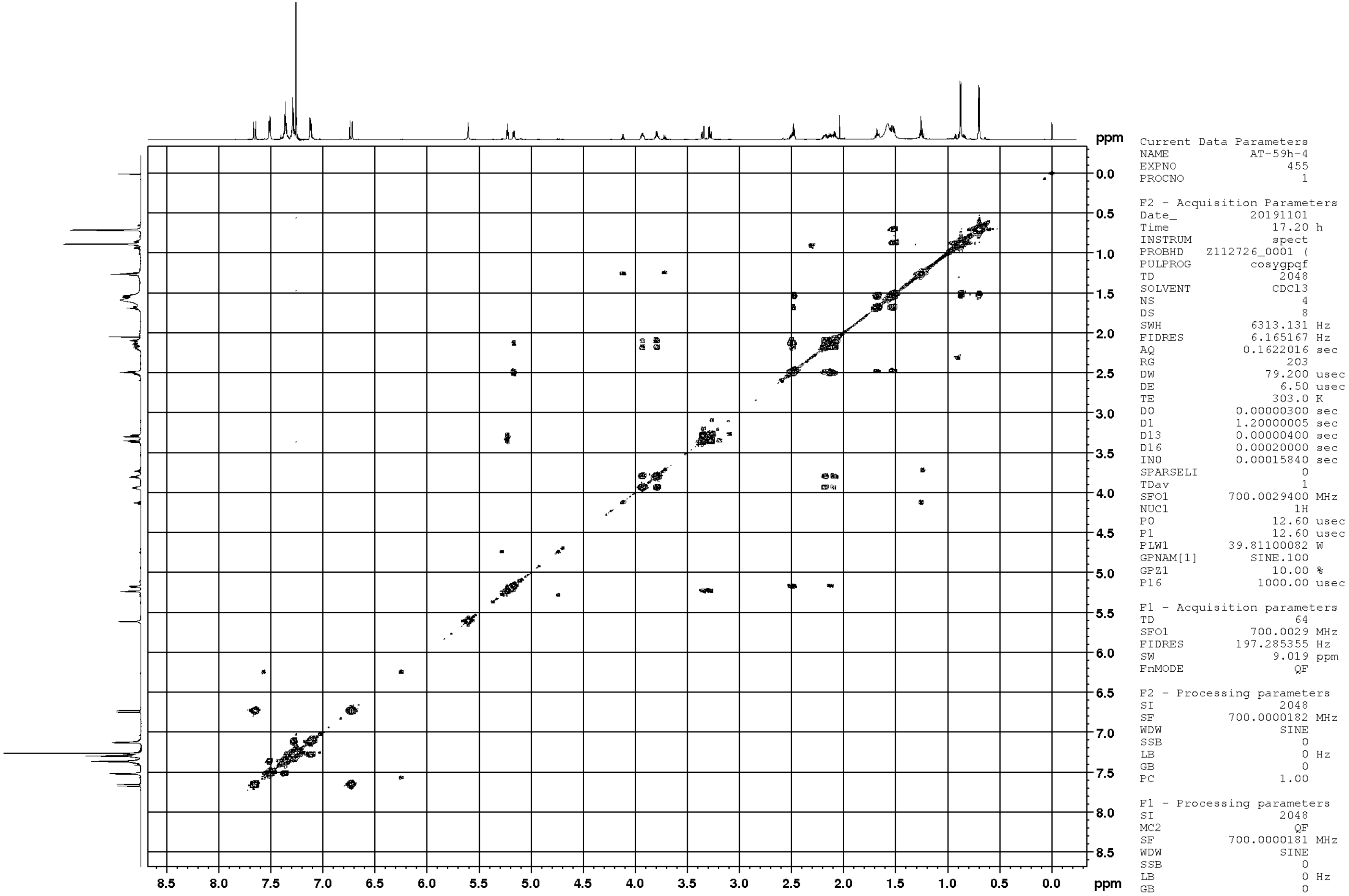

Figure S15. NOESY spectrum of asterripeptide B (2)

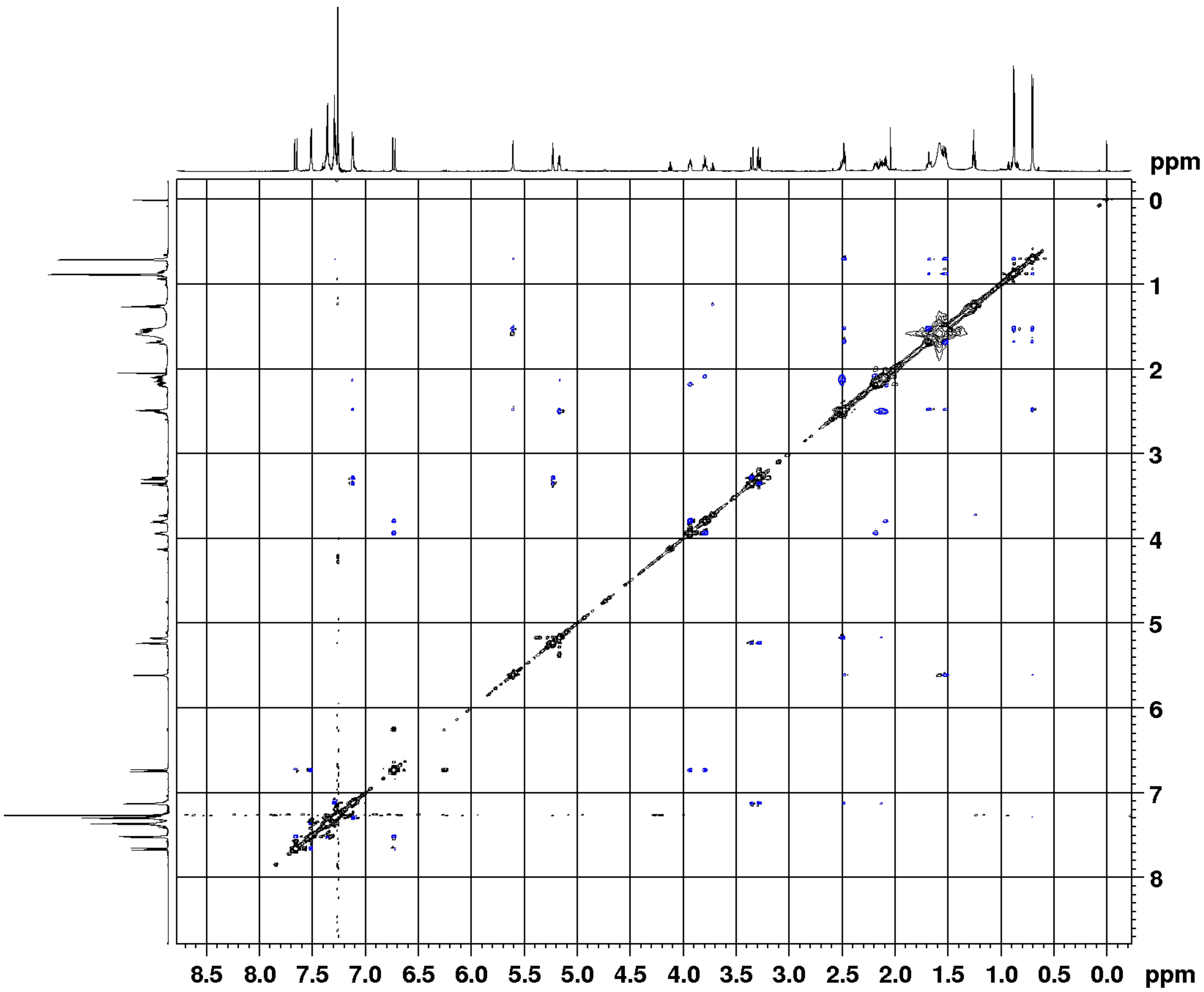


```
Current Data Parameters
NAME            AT-59h-4
EXPNO               6030
PROCNO                 1

F2 - Acquisition Parameters
Date_           20191101
Time               17.28 h
INSTRUM            spect
PROBHD   Z112726_0001 (
PULPROG        noesygpph
TD                  2048
SOLVENT            CDCl3
NS                    16
DS                    16
SWH             6313.131 Hz
FIDRES          6.165167 Hz
AQ             0.1622016 sec
RG                   203
DW                79.200 usec
DE                  6.50 usec
TE                 303.0 K
D0            0.00006316 sec
D1            1.00000000 sec
D8            1.00000000 sec
D16           0.00020000 sec
IN0           0.00015840 sec
TDav                   1
SFO1         700.0030100 MHz
NUC1                  1H
P1                 12.60 usec
P2                 25.20 usec
PLW1         39.81100082 W
GPNAM[1]        SINE.100
GPZ1               20.00 %
P16              1000.00 usec

F1 - Acquisition parameters
TD                   256
SFO1             700.003 MHz
FIDRES         49.321339 Hz
SW                 9.019 ppm
FnMODE              TPPI

F2 - Processing parameters
SI                  2048
SF           700.0000182 MHz
WDW                QSINE
SSB                    2
LB                     0 Hz
GB                     0
PC                  1.00

F1 - Processing parameters
SI                  2048
MC2                 TPPI
SF           700.0000168 MHz
```

Figure S16. MS and MS/MS data of asterripeptide B (2)

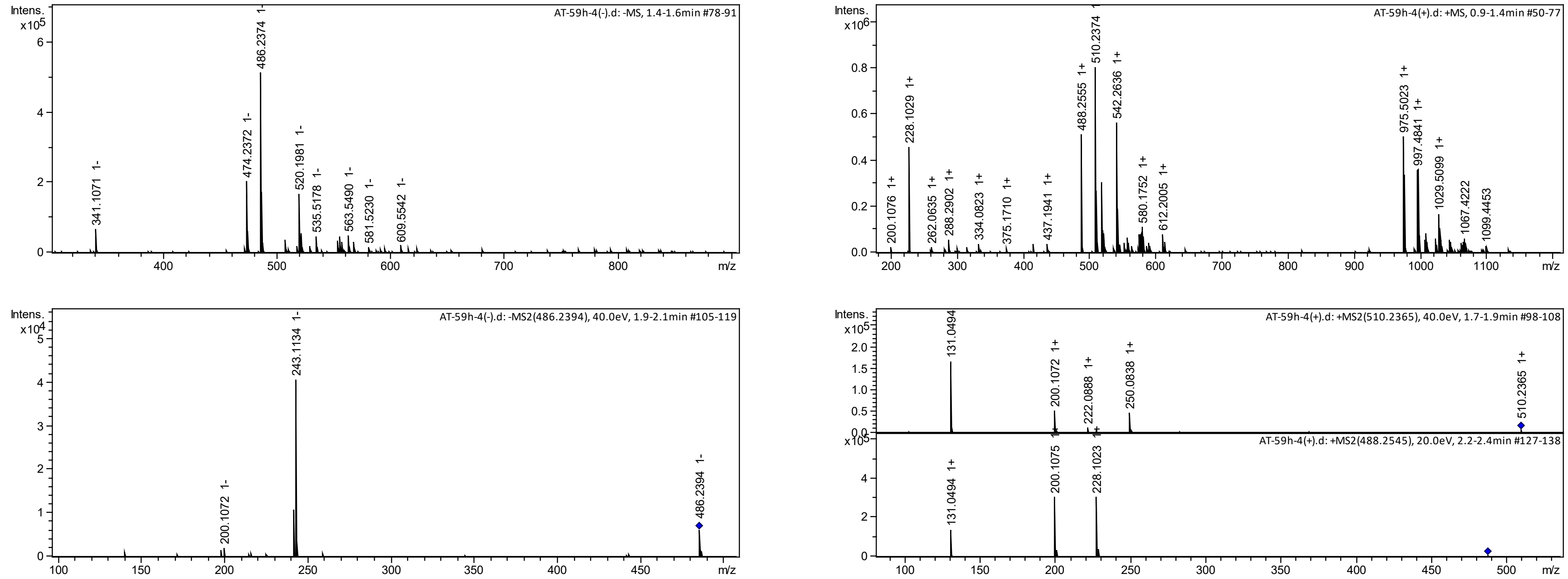

	meas.	calc.	Δ (ppm)
$[M-H]^-$	486,2374	486,2398	5,1
$[M+Na]^+$	510,2374	510,2363	-2,1

Figure S17. ¹H NMR spectrum of asterripeptide C (3)

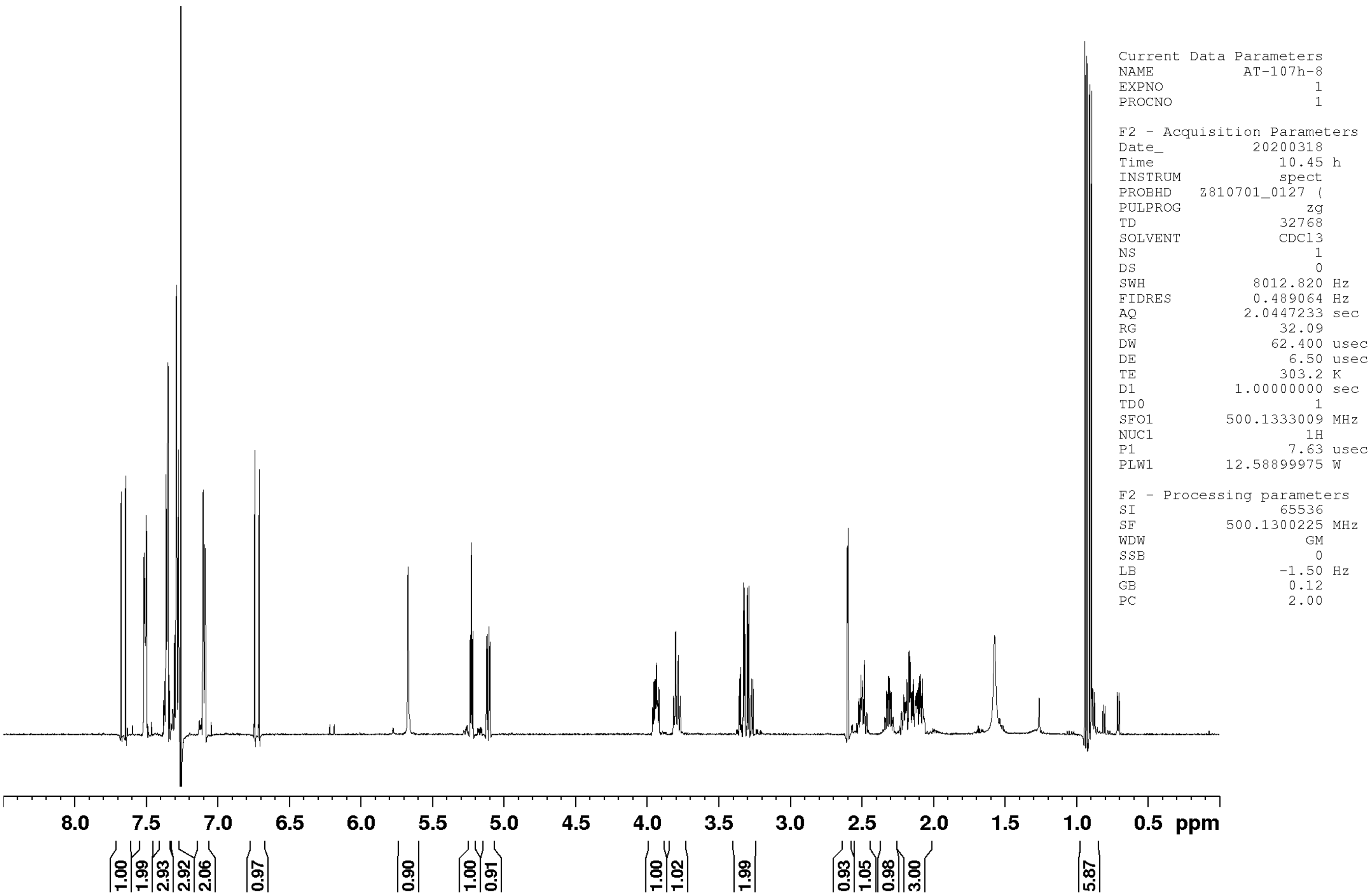

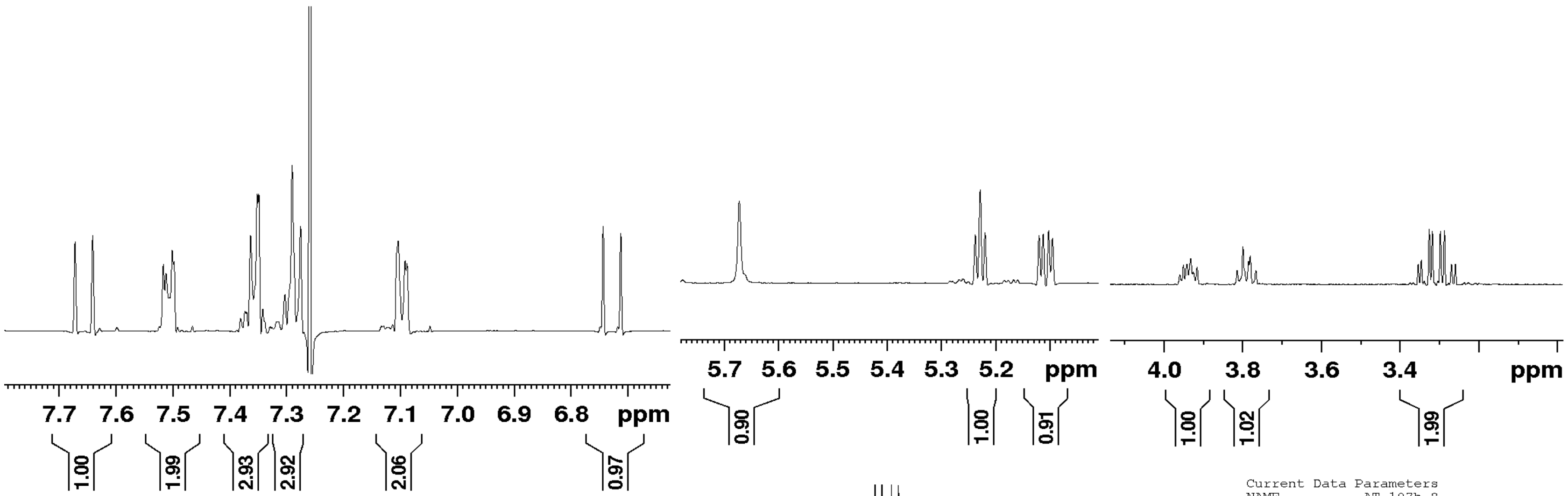

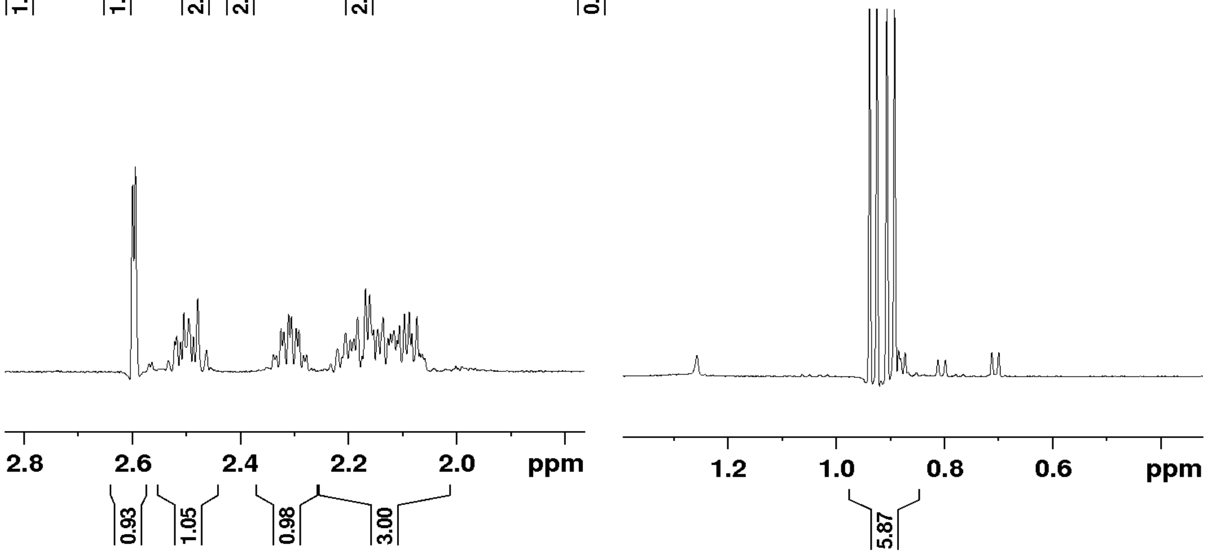


```
Current Data Parameters
NAME             AT-107h-8
EXPNO                    1
PROCNO                   1

F2 - Acquisition Parameters
Date_             20200318
Time                 10.45 h
INSTRUM              spect
PROBHD   Z810701_0127 (
PULPROG                 zg
TD                   32768
SOLVENT              CDCl3
NS                       1
DS                       0
SWH            8012.820 Hz
FIDRES         0.489064 Hz
AQ            2.0447233 sec
RG                   32.09
DW                  62.400 usec
DE                    6.50 usec
TE                   303.2 K
D1           1.00000000 sec
TD0                      1
SFO1        500.1333009 MHz
NUC1                    1H
P1                    7.63 usec
PLW1        12.58899975 W

F2 - Processing parameters
SI                   65536
SF          500.1300225 MHz
WDW                     GM
SSB                      0
LB                   -1.50 Hz
GB                    0.12
PC                    2.00
```

Figure S18. ^{13}C NMR spectrum of asterripeptide C (3)

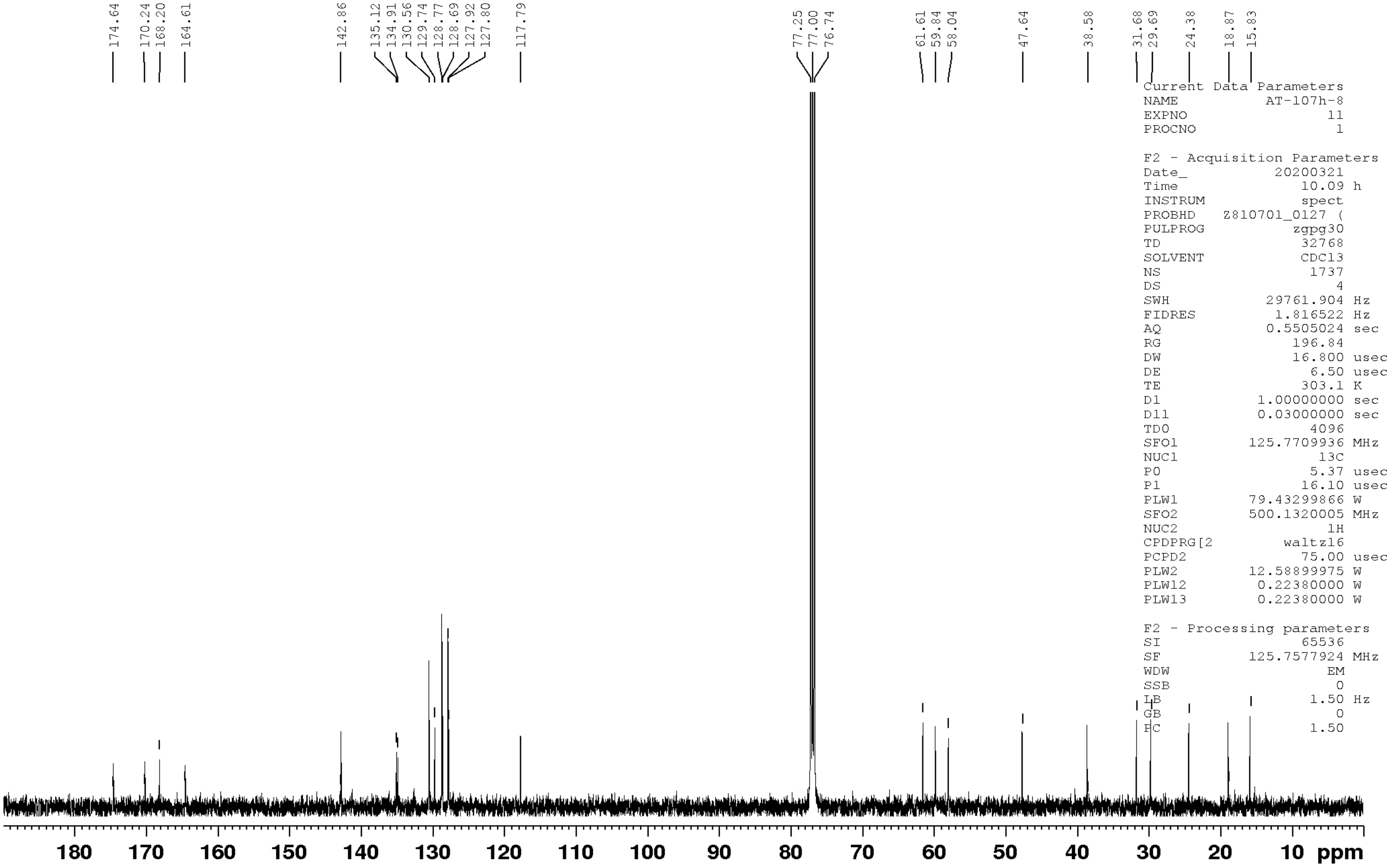

Figure S19. DEPT NMR spectrum of asterripeptide C (3)

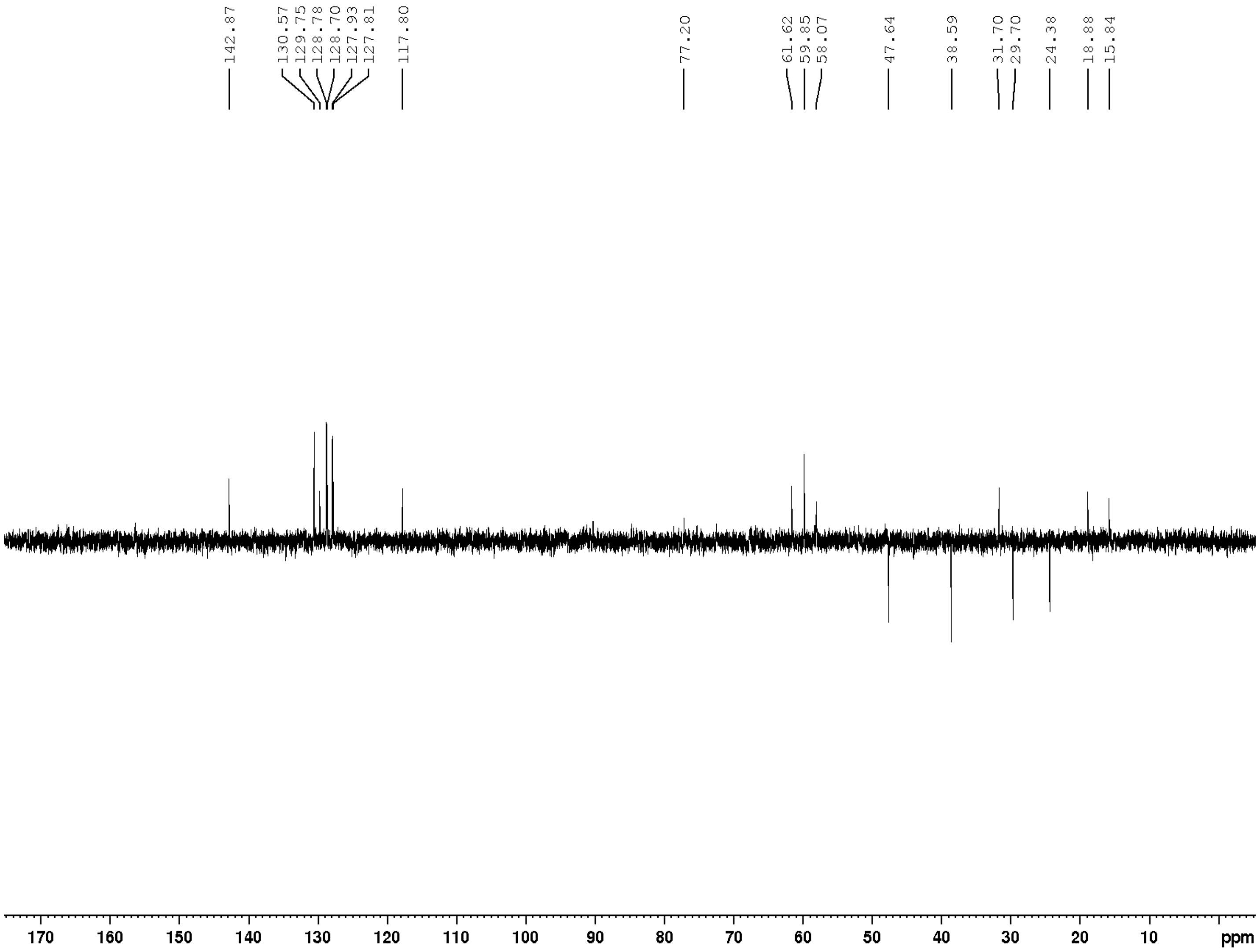


```
Current Data Parameters
NAME          AT-107h-8
EXPNO                75
PROCNO                1

F2 - Acquisition Parameters
Date_          20200322
Time               7.53 h
INSTRUM           spect
PROBHD   Z810701_0127 (
PULPROG       deptsp135
TD                32768
SOLVENT           CDCl3
NS                26065
DS                    2
SWH           22727.273 Hz
FIDRES         1.387163 Hz
AQ            0.7208960 sec
RG               196.84
DW               22.000 usec
DE                 6.50 usec
TE                303.2 K
CNST2       145.0000000
D1           1.00000000 sec
D2           0.00344828 sec
D12          0.00002000 sec
TD0                4096
SFO1        125.7684784 MHz
NUC1                13C
P1                16.10 usec
P13             2000.00 usec
PLW0                  0 W
PLW1        79.43299866 W
SPNAM[5]    Crp60comp.4
SPOAL5            0.500
SPOFFS5               0 Hz
SPW5        31.45899963 W
SFO2        500.1320005 MHz
NUC2                 1H
CPDPRG[2        waltz16
P3                10.50 usec
P4                21.00 usec
PCPD2             75.00 usec
PLW2        12.58899975 W
PLW12        0.24674000 W

F2 - Processing parameters
SI                65536
SF          125.7577911 MHz
WDW                  EM
SSB                   0
LB                 1.50 Hz
GB                    0
PC                 1.20
```

Figure S20. HSQC NMR spectrum of asterripeptide C (3)

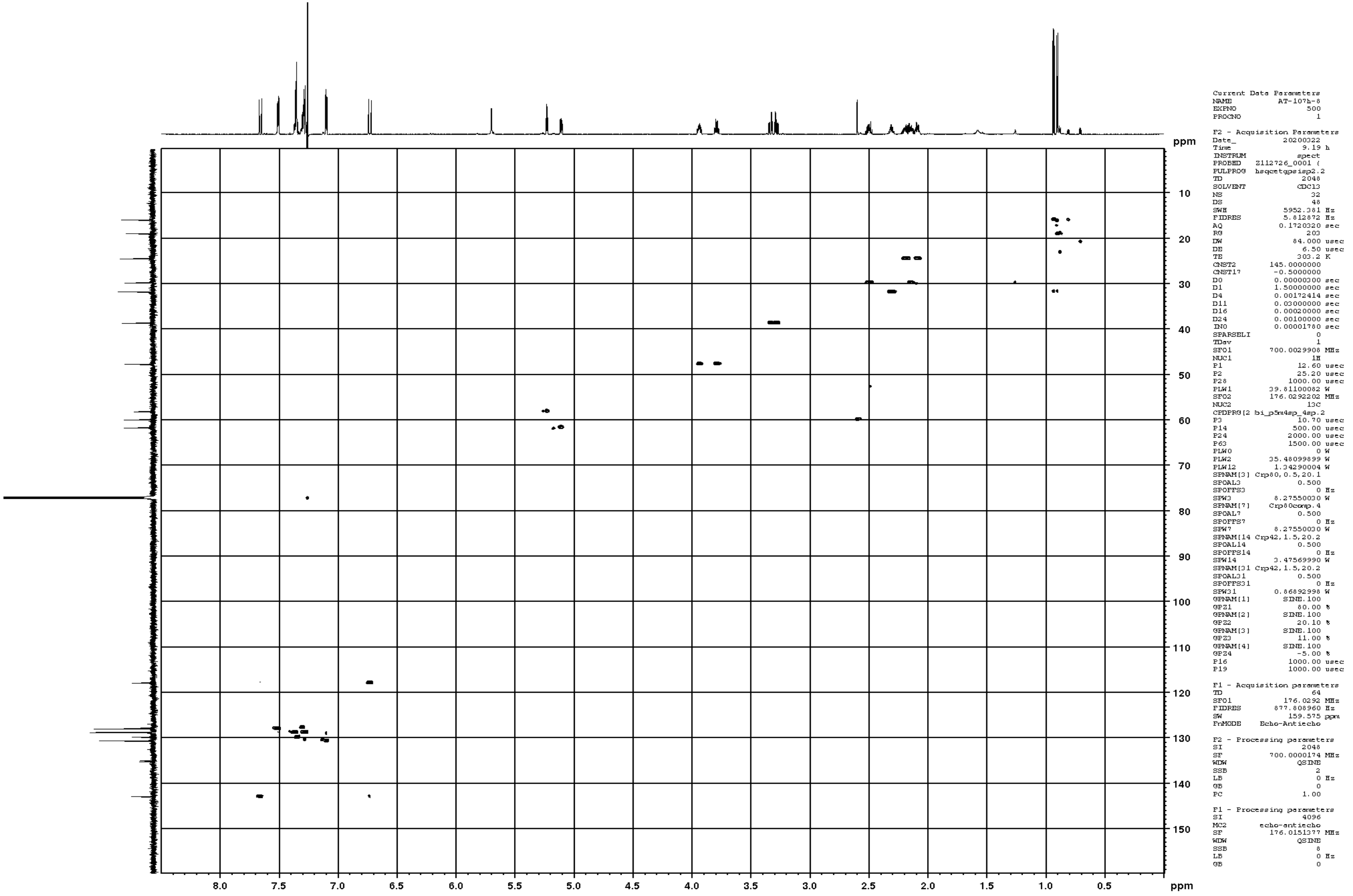

Figure S21. HMBC NMR spectrum of asterripeptide C (3)

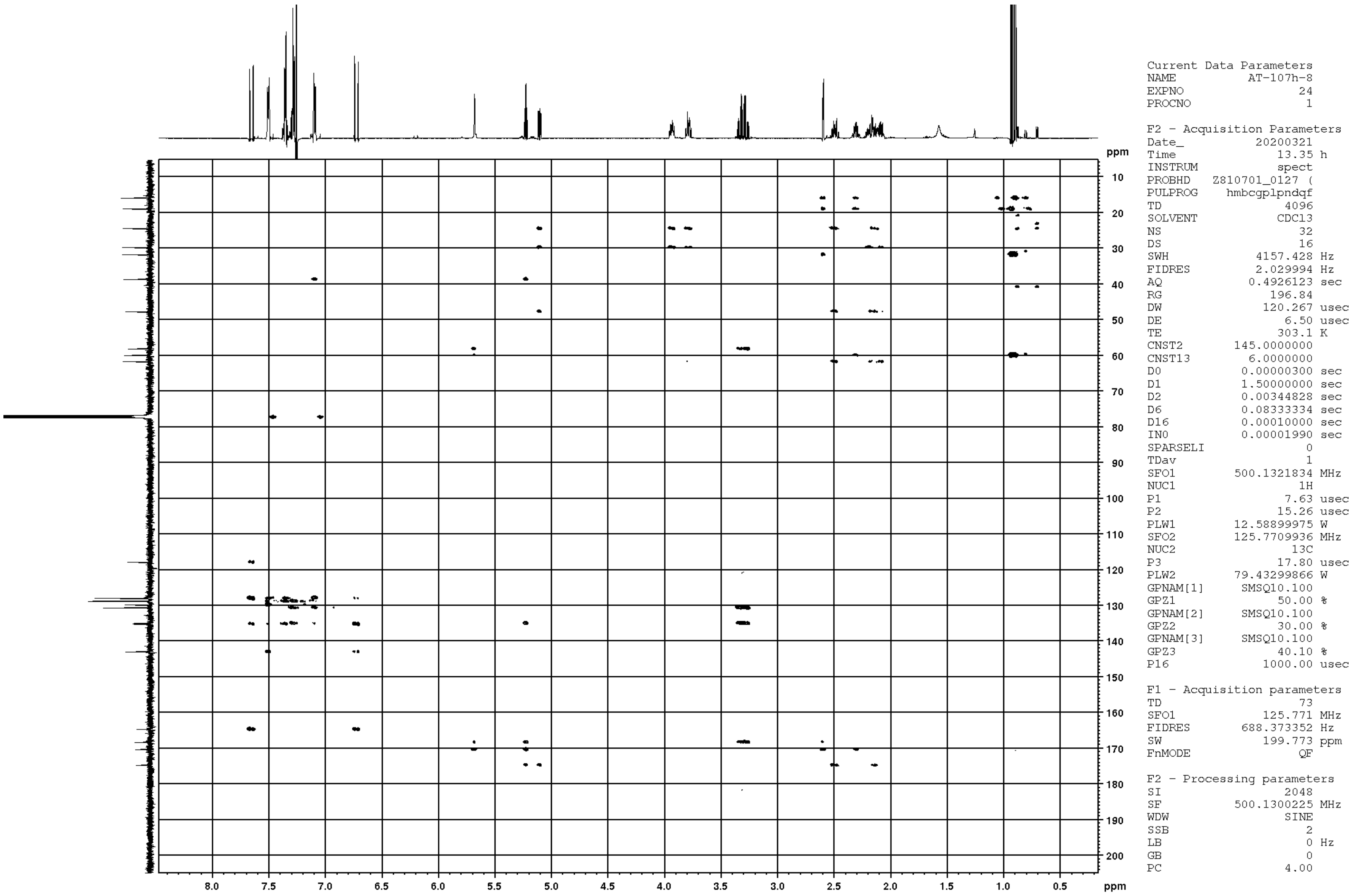

Figure S22. ^1H-^1H COSY NMR spectrum of asterripeptide C (3)

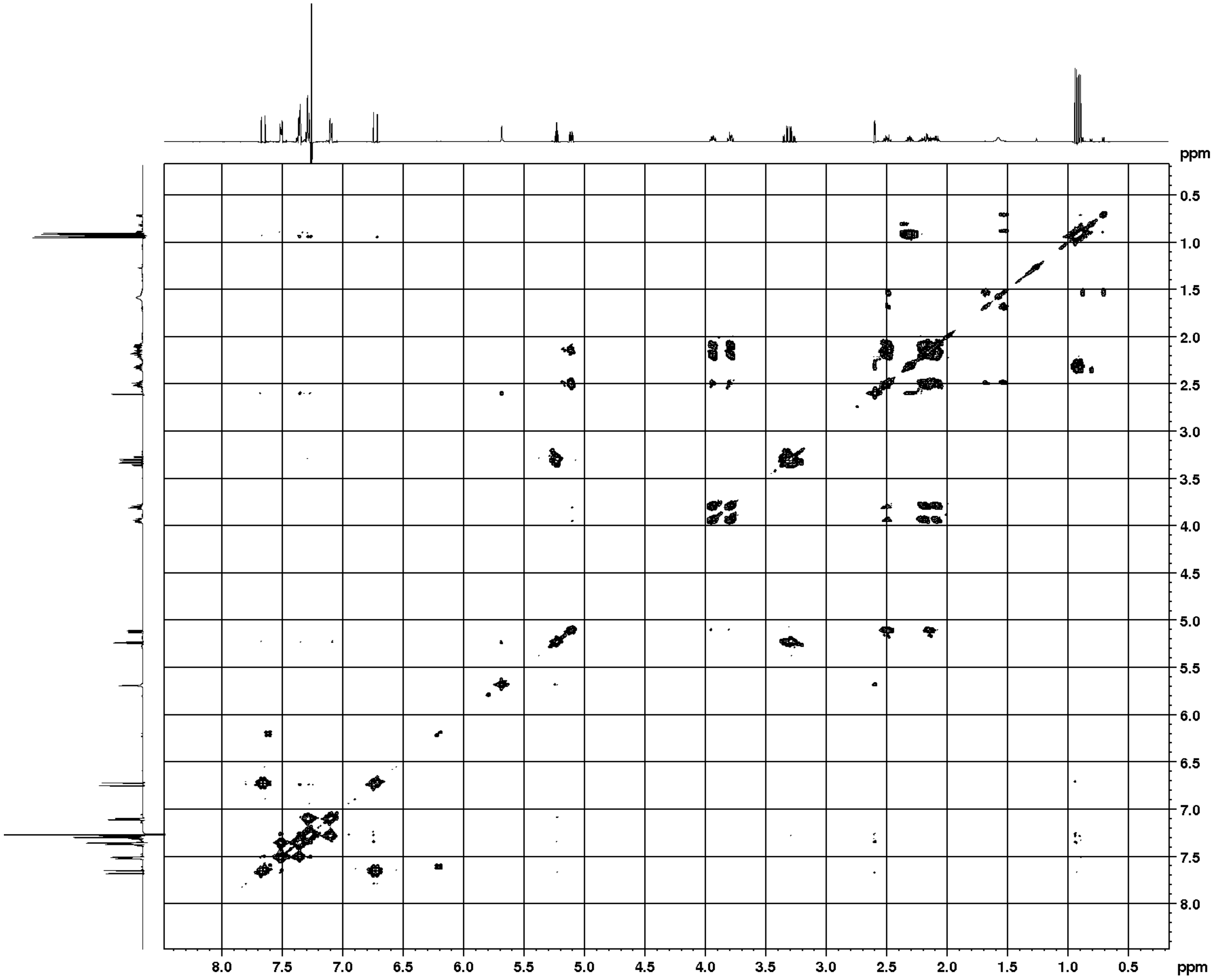


```
Current Data Parameters
NAME            AT-107h-8
EXPNO                  21
PROCNO                  1

F2 - Acquisition Parameters
Date_            20200321
Time                10.43 h
INSTRUM             spect
PROBHD   Z810701_0127 (
PULPROG          cosyqf45
TD                   2048
SOLVENT             CDCl3
NS                      4
DS                     16
SWH              4157.428 Hz
FIDRES           4.059988 Hz
AQ              0.2463061 sec
RG                  91.04
DW                120.267 usec
DE                   6.50 usec
TE                  303.2 K
D0             0.00000300 sec
D1             2.00000000 sec
IN0            0.00024060 sec
SPARSELI                0
TDav                    1
SFO1          500.1321834 MHz
NUC1                   1H
P1                   7.63 usec
PLW1          12.58899975 W

F1 - Acquisition parameters
TD                    128
SFO1             500.1324 MHz
FIDRES          64.941811 Hz
SW                  8.310 ppm
FnMODE                 QF

F2 - Processing parameters
SI                   2048
SF            500.1300225 MHz
WDW                  SINE
SSB                     0
LB                      0 Hz
GB                      0
PC                   4.00

F1 - Processing parameters
SI                   2048
MC2                    QF
SF            500.1301891 MHz
WDW                  SINE
SSB                     0
LB                      0 Hz
GB                      0
```

Figure S23. ROESY NMR spectrum of asterripeptide C (3)

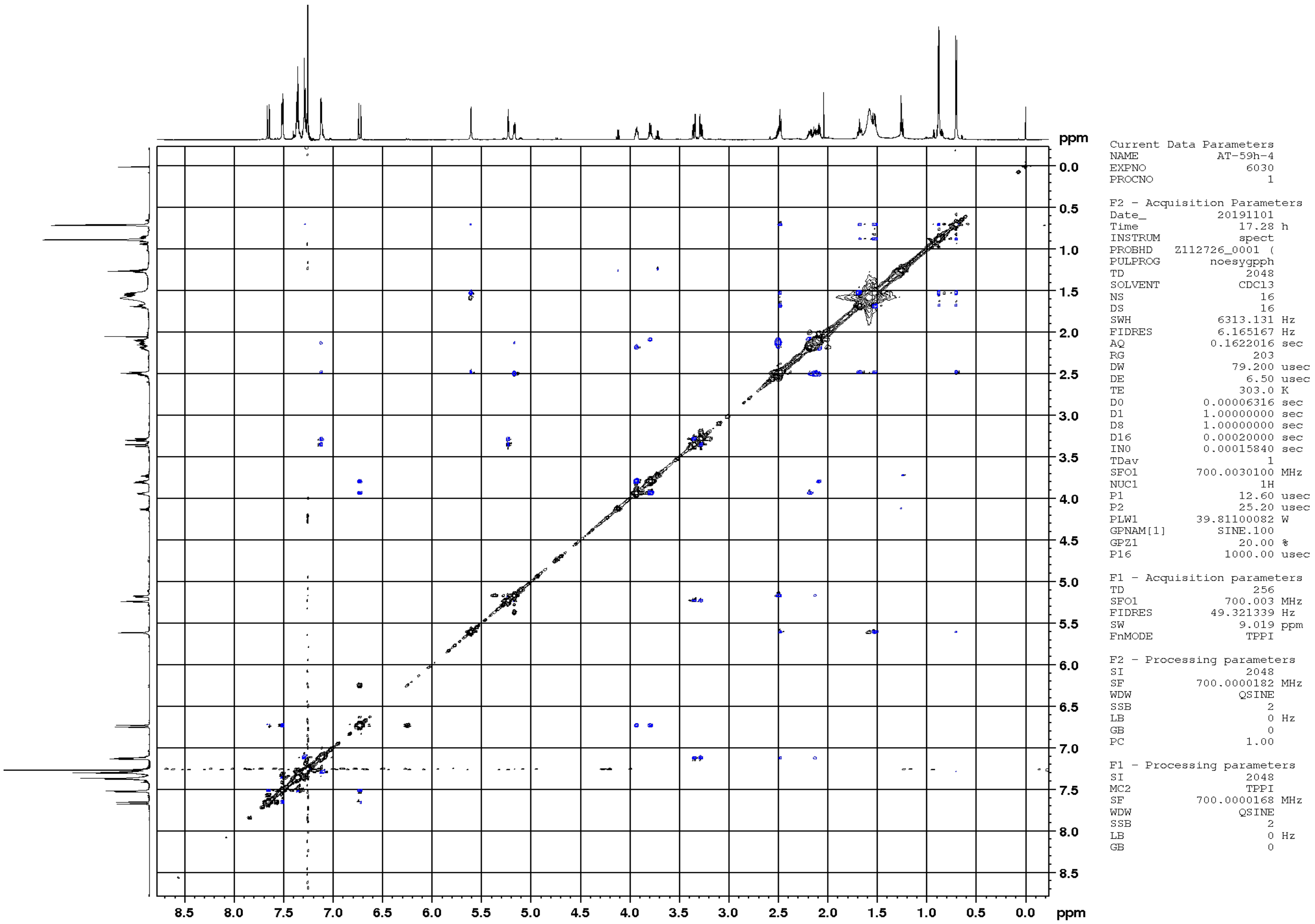

Figure S24. MS and MS/MS data of asterripeptide C (3)

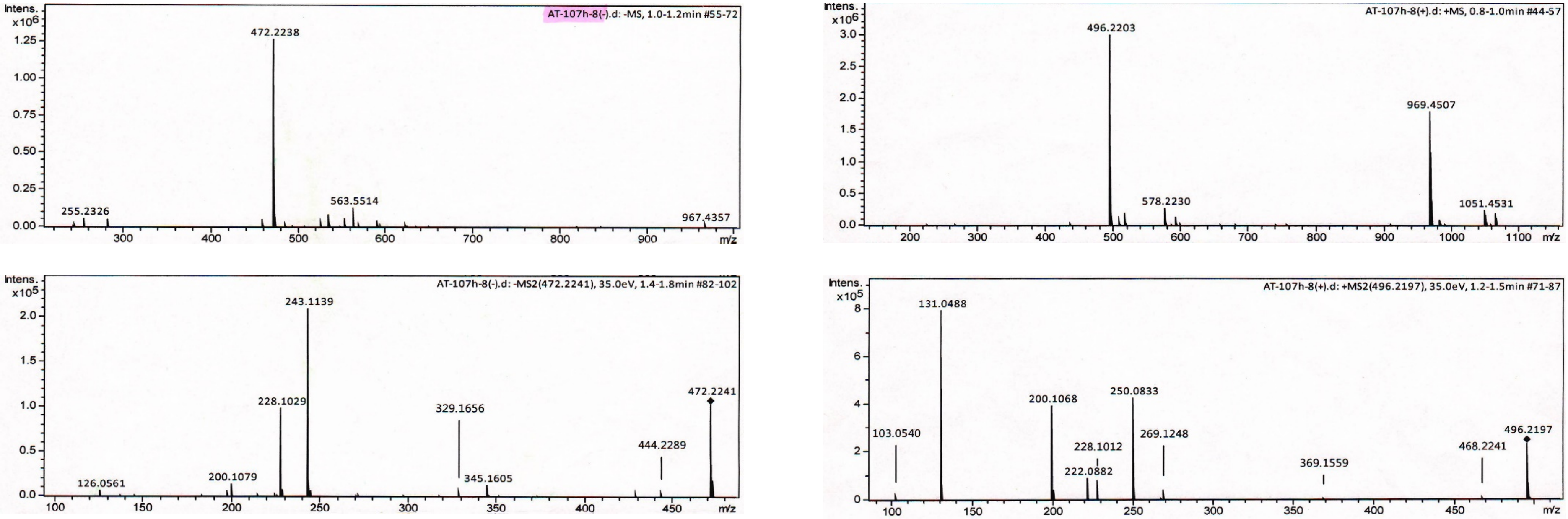

	meas.	calc.	Δ (ppm)
[M-H]-	472.2238	472.2242	0.7
[M+Na]+	496.2203	496.2207	0.8

Figure S25. HPLC profile of *L*-FDAA-derivatives of asterripeptide A (1) hydrolysis products (HP)

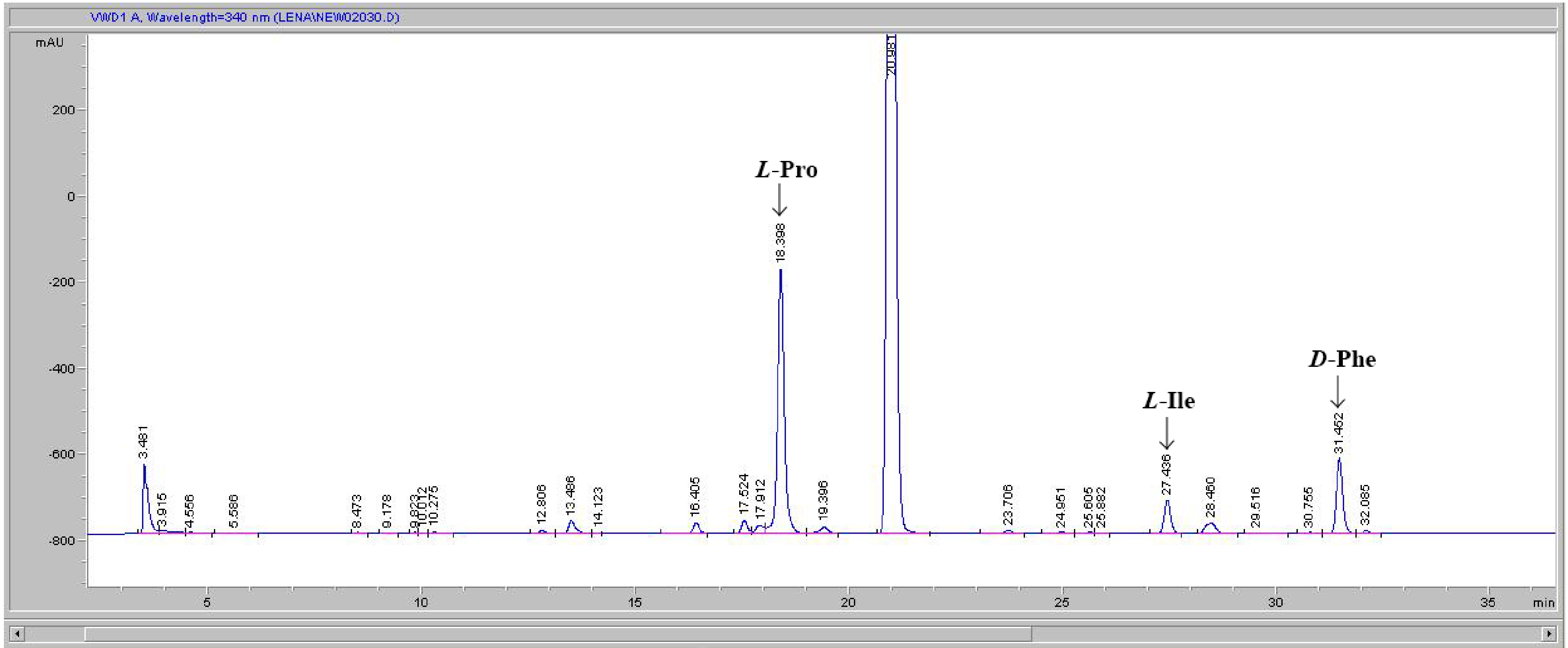

File Information	
LC-File	NEW02030.D
File Path	C:\HPCHEM\1\DATA\LENA\
Date	23-Dec-21, 11:32:13
Sample	AT-59h-1
Sample Info	
Barcode	
Operator	Rimma
Method	MURFY.M
Analysis Time	54.983 min
Sampling Rate	0.0049 min (0.294 sec), 11330 datapoints

#	Time	Area	Height	Width	Symmetry
1	3.481	1447.8	163.6	0.1164	0.233
2	3.915	156.4	6.9	0.2819	0.145
3	4.556	60.8	3	0.2587	0.291
4	5.586	55.1	2	0.3406	1.728
5	8.473	20.1	2.4	0.1255	0.83
6	9.178	18.7	1.7	0.1573	0.622
7	9.823	25.1	3	0.1278	0.911
8	10.012	18.9	2.2	0.1262	0.869
9	10.275	43.2	3.9	0.1594	0.543
10	12.806	81.6	7.3	0.1604	1.062
11	13.486	340.7	29.9	0.1679	0.664
12	14.123	17.4	1.9	0.1321	0.968
13	16.405	273.3	25.9	0.1565	1.035
14	17.524	291.8	30.1	0.148	0.866
15	17.912	259	18.5	0.2083	1.191
16	18.398	6653	614.4	0.1627	0.918

Figure S26. HPLC profile of *L*-FDAA-derivatives of asterripeptide A (1) HP (a), asterripiptide A HP+*L*-Ile (b) and asterripiptide A HP+*D*,*L*-Ile (c)

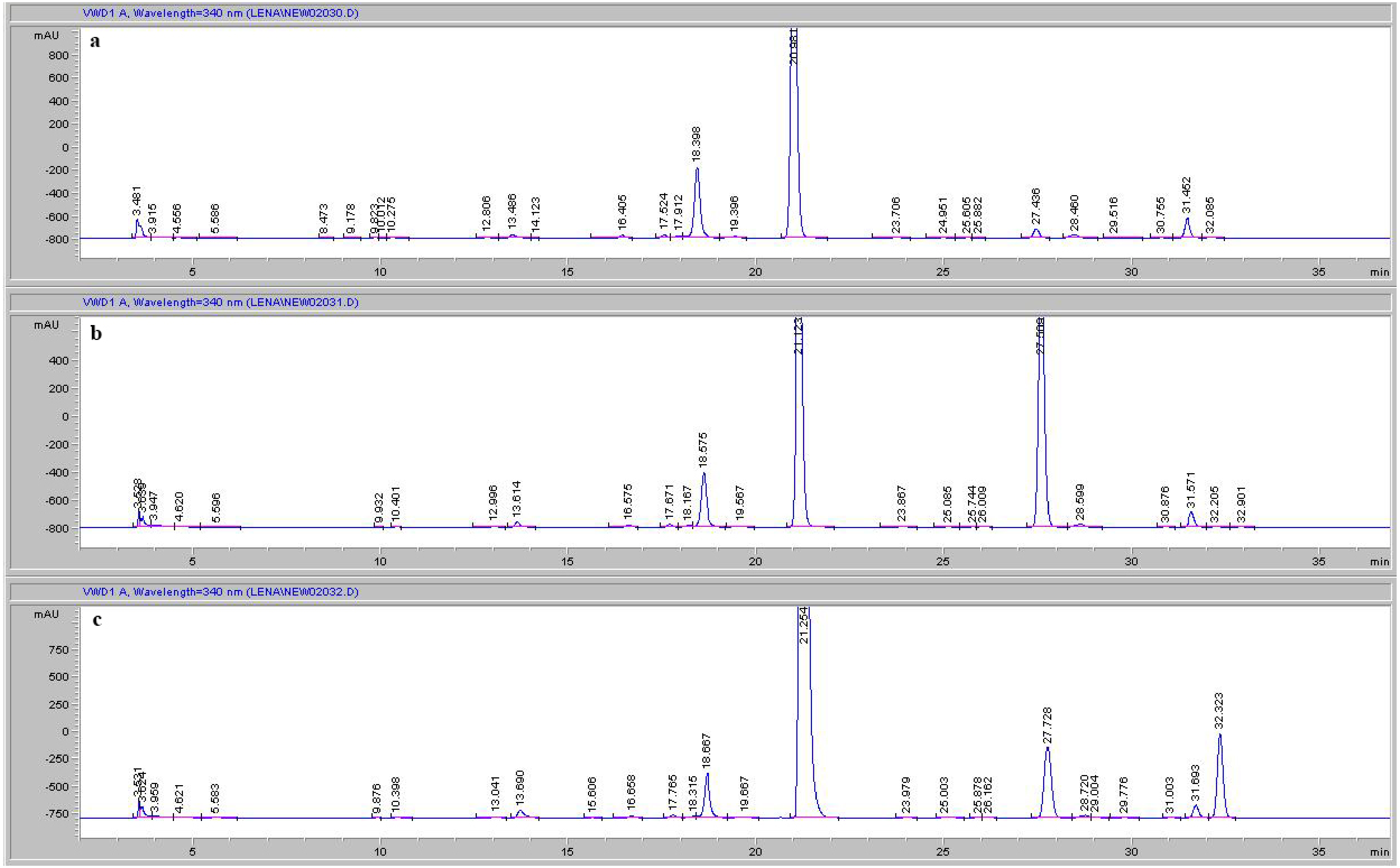

Figure S27. HPLC profile of *L*-FDAA-derivatives of asterripeptide A (1) HP, asterripiptide A HP+*L*-Phe and asterripiptide A HP+*D*,*L*-Phe

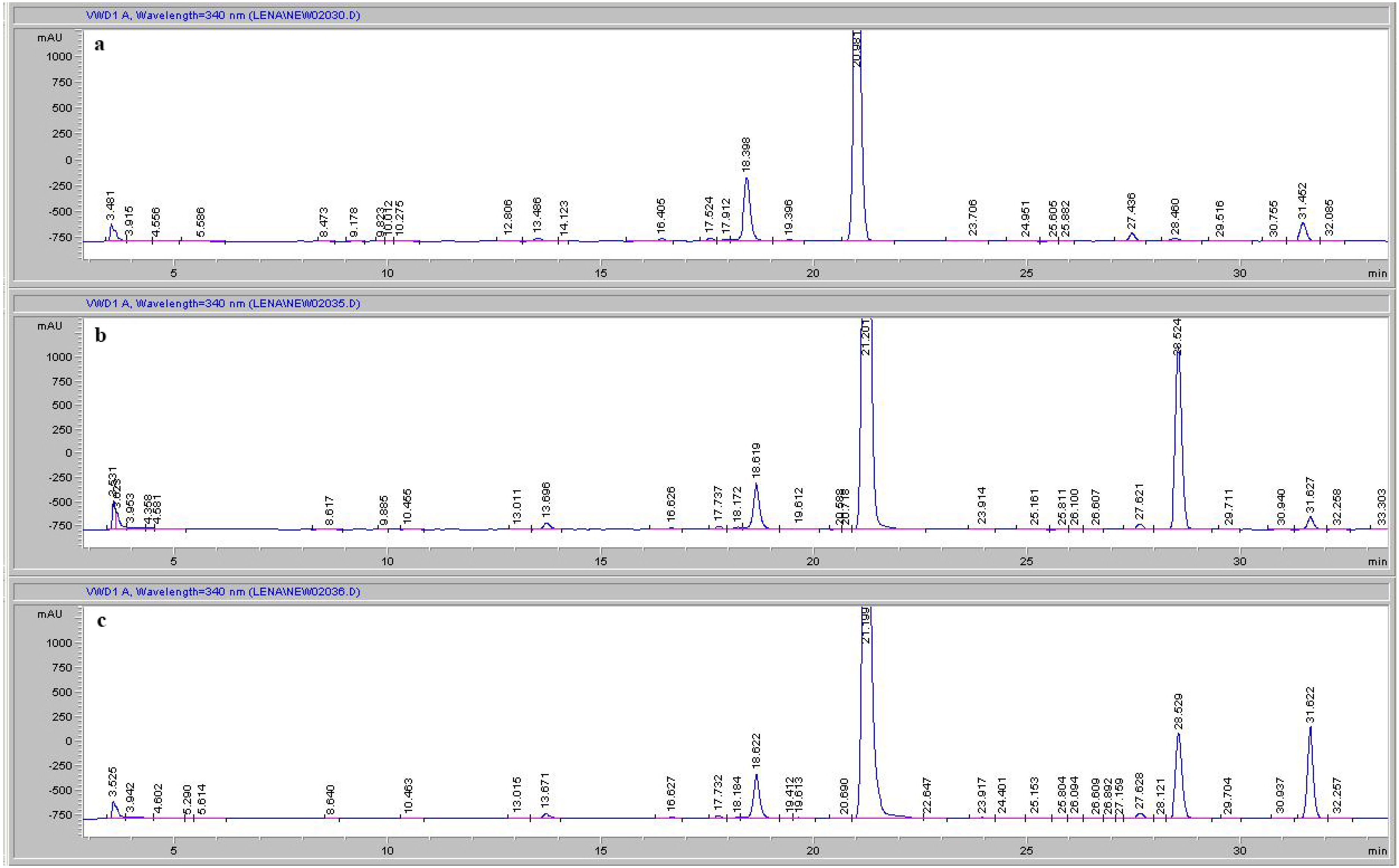

Figure S28. HPLC profile of *L*-FDAA-derivatives of asterripeptide A (1) HP, asterripiptide A HP+*D*,*L*-Pro and asterripiptide A HP+*L*-Pro

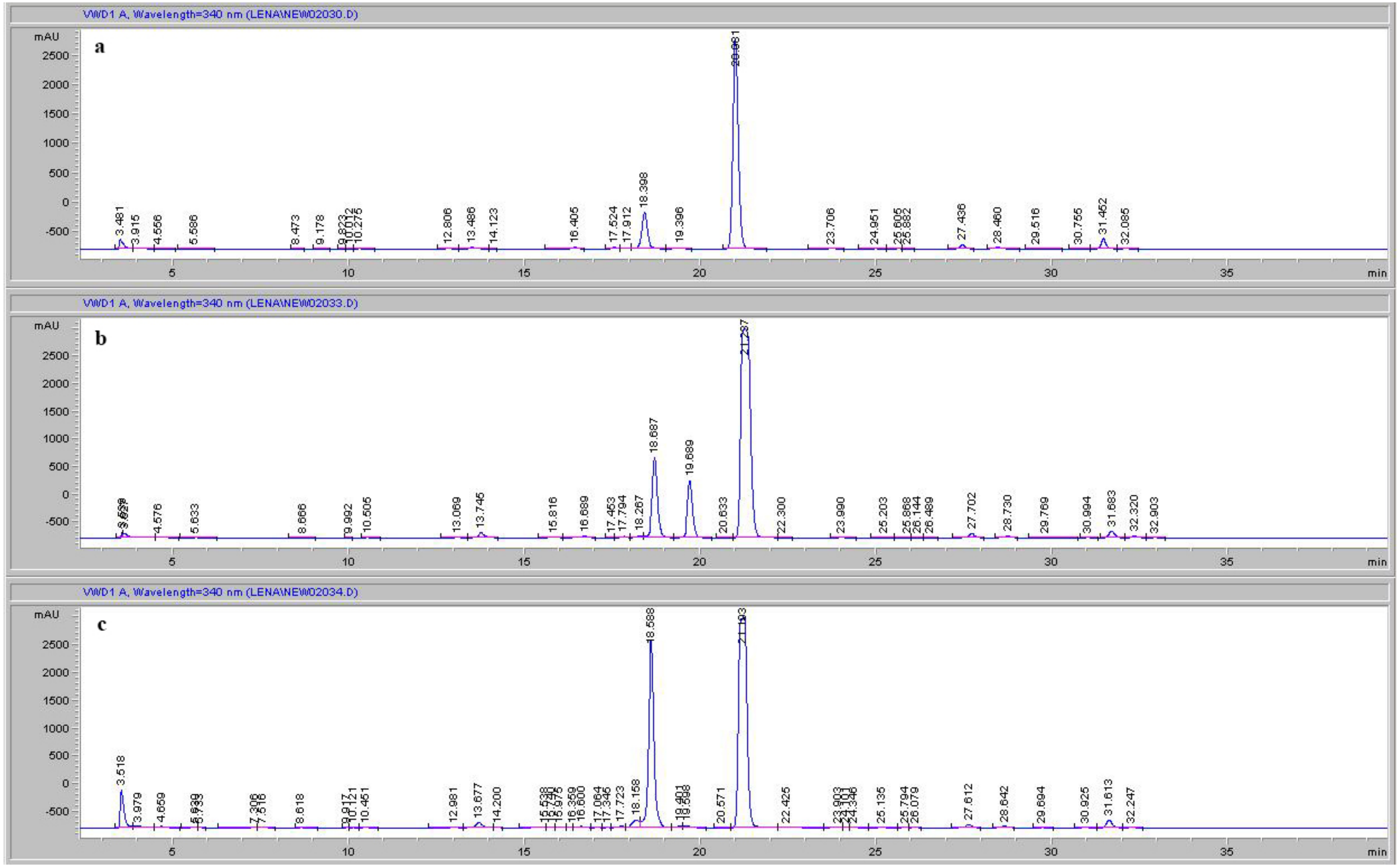

Figure S29. HPLC profile of *L*-FDAA-derivatives of asterripeptide B (2) HP

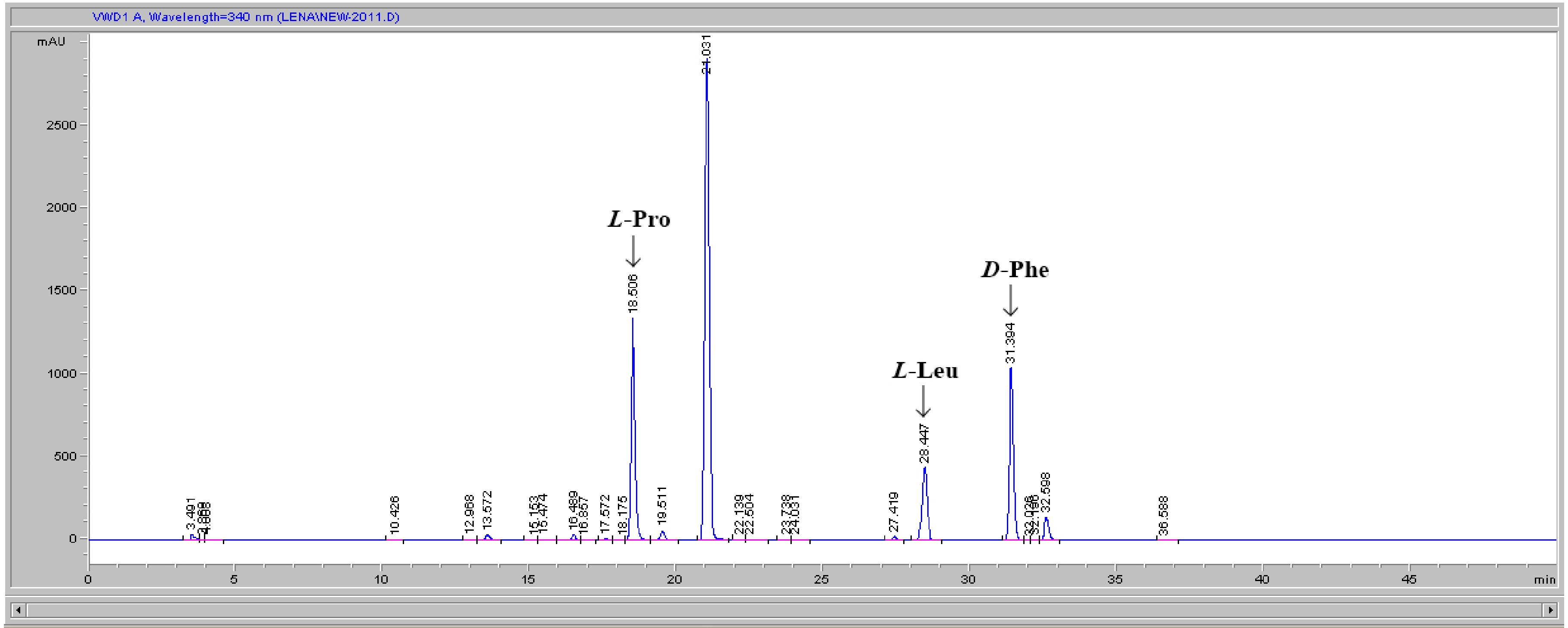

File Information

LC-File	NEW-2011.D
File Path	C:\HPCHEM\1\DATA\LENA\
Date	12-Oct-21, 13:02:18
Sample	Sample-1_new
Sample Info	
Barcode	
Operator	Rimma
Method	MURFY.M
Analysis Time	49.984 min
Sampling Rate	0.0049 min (0.294 sec), 10300 datapoints

#	Time	Area	Height	Width	Symmetry
1	3.491	332.2	37.8	0.1228	0.331
2	3.869	46.6	4.7	0.1361	1.175
3	4.008	88.4	4.2	0.2628	0.17
4	10.426	22.5	2.4	0.1365	1.089
5	12.968	61.6	4.8	0.2038	1.062
6	13.572	316.9	31.9	0.1478	0.789
7	15.153	34.9	2.1	0.2217	1.776
8	15.474	80.5	4.5	0.2431	0.472
9	16.489	312.9	30.9	0.1531	0.89
10	16.857	22.2	1.8	0.1771	0.686
11	17.572	87.1	8.6	0.1559	0.909
12	18.175	24.4	1.9	0.1834	2.265
13	18.506	12902.5	1341.3	0.1472	0.819
14	19.511	563.2	55.2	0.1524	0.892
15	21.031	30371.8	2920.2	0.1607	0.864

Figure S30. HPLC profiles of *L*-FDAA-derivatives of asterripeptide B (2) HP (a), asterripiptide B HP+*L*-Leu (b) and asterripiptide B HP+*D*,*L*-Leu (c)

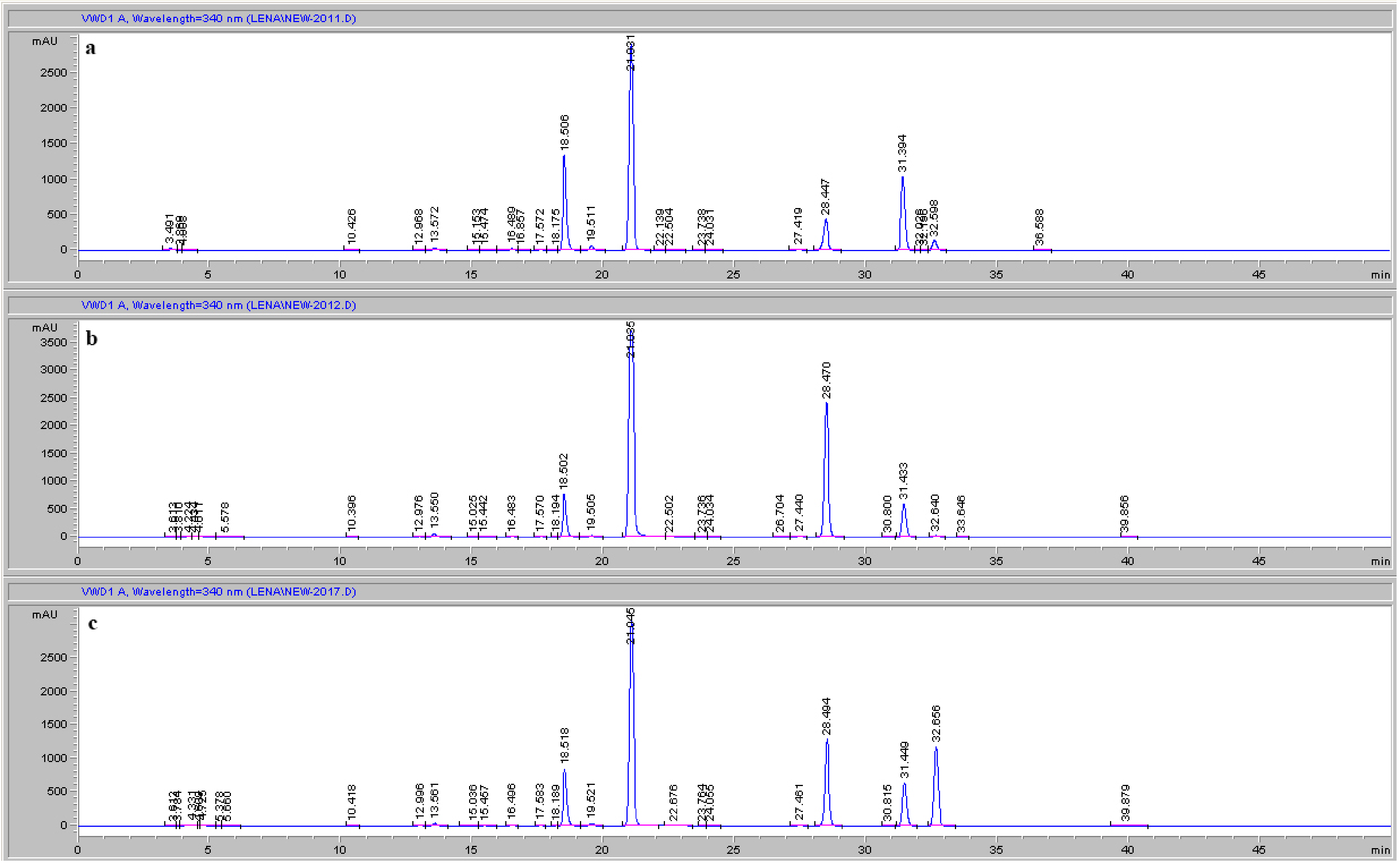

Figure S31. HPLC profiles of *L*-FDAA-derivatives of asterripeptide B (2) HP (a), asterripiptide B+ *D*,*L*-Pro (b) and asterripiptide B+ *L*-Pro (c)

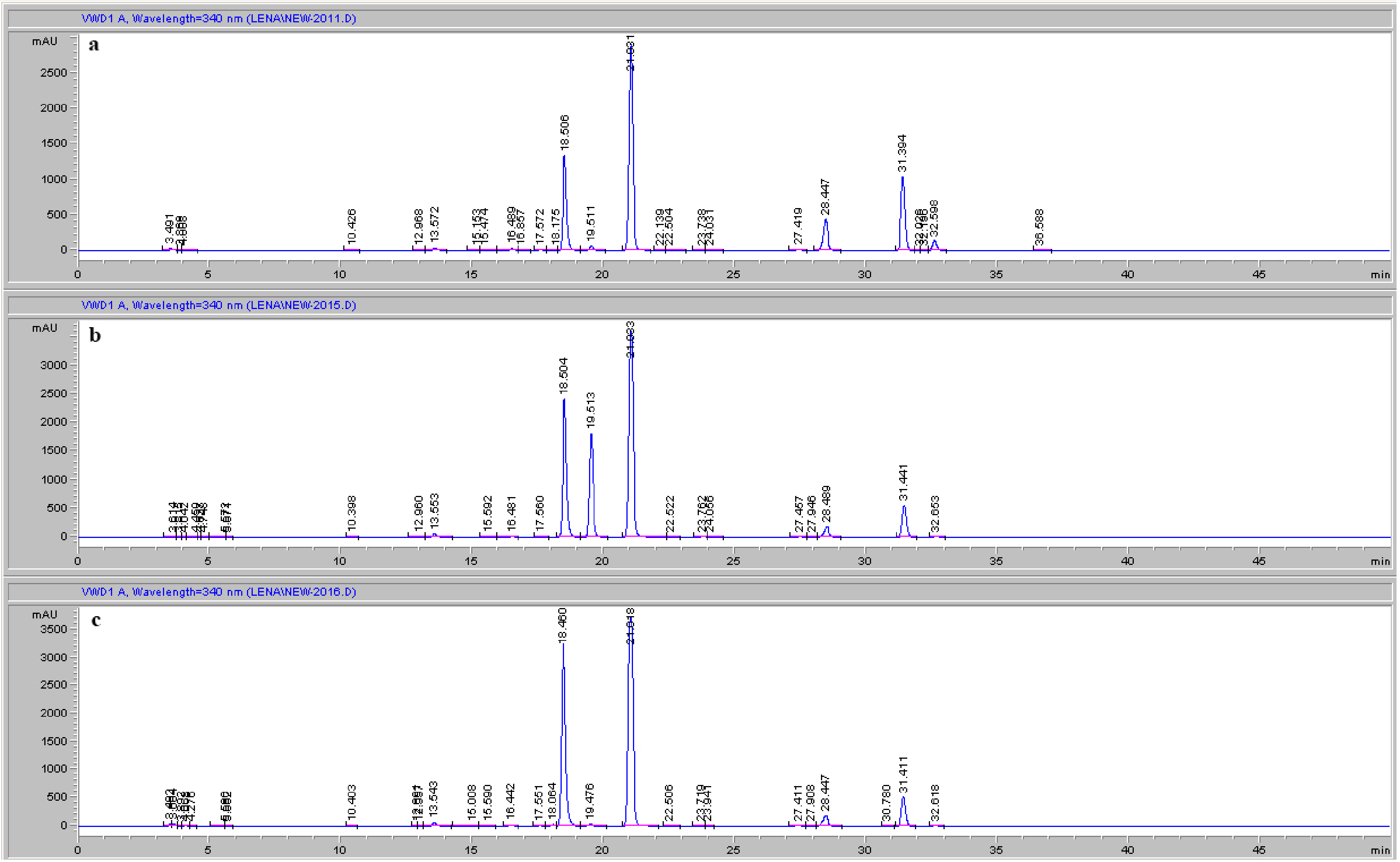

Figure S32. HPLC profiles of *L*-FDAA-derivatives of asterripeptide B (2) HP (a), asterripiptide B HP+ *L*-Phe (b) and asterripiptide B HP+ *D,L*-Phe (c)

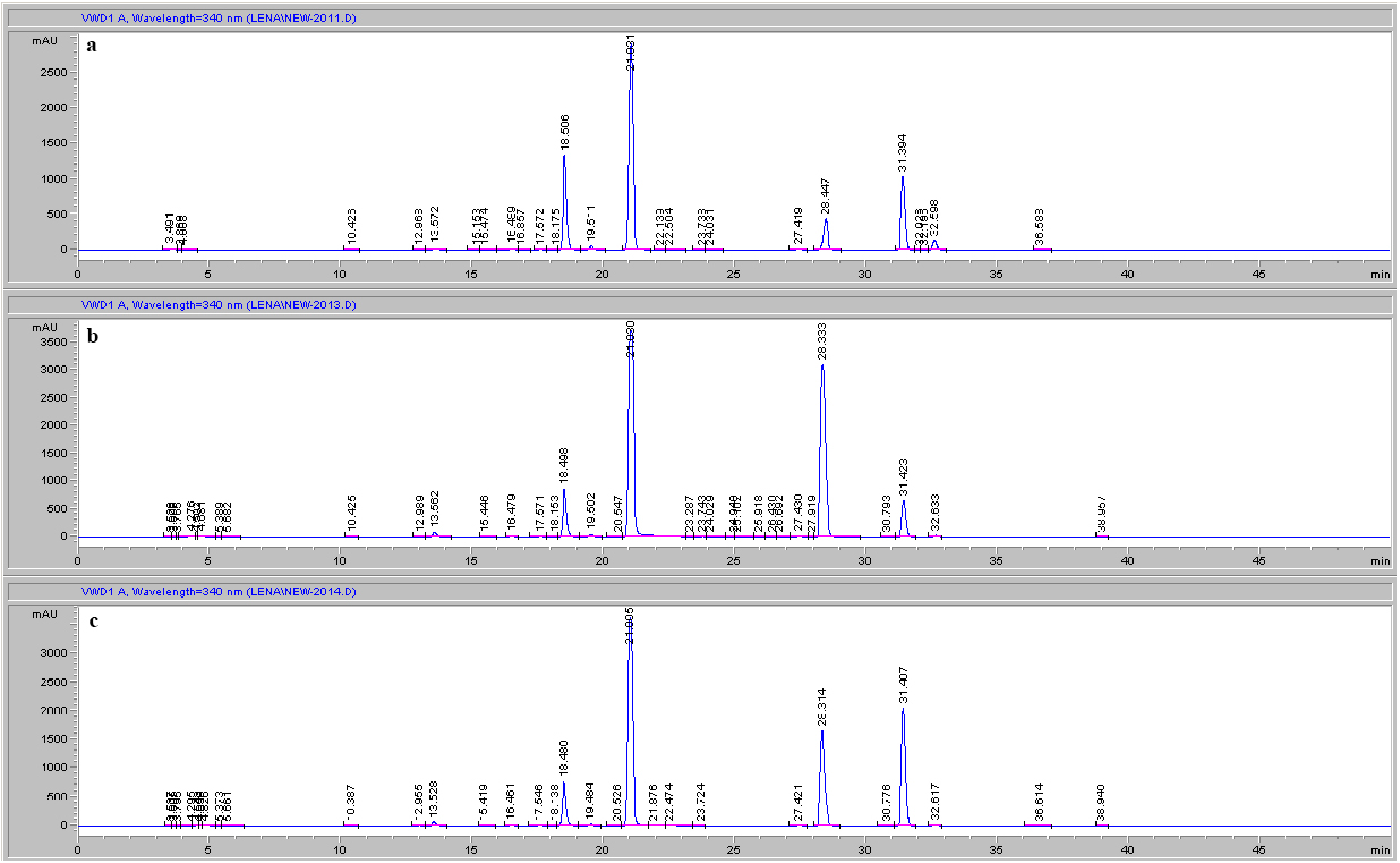

Figure S33. HPLC profile of *L*-FDAA-derivatives of asterripeptide C (3) HP

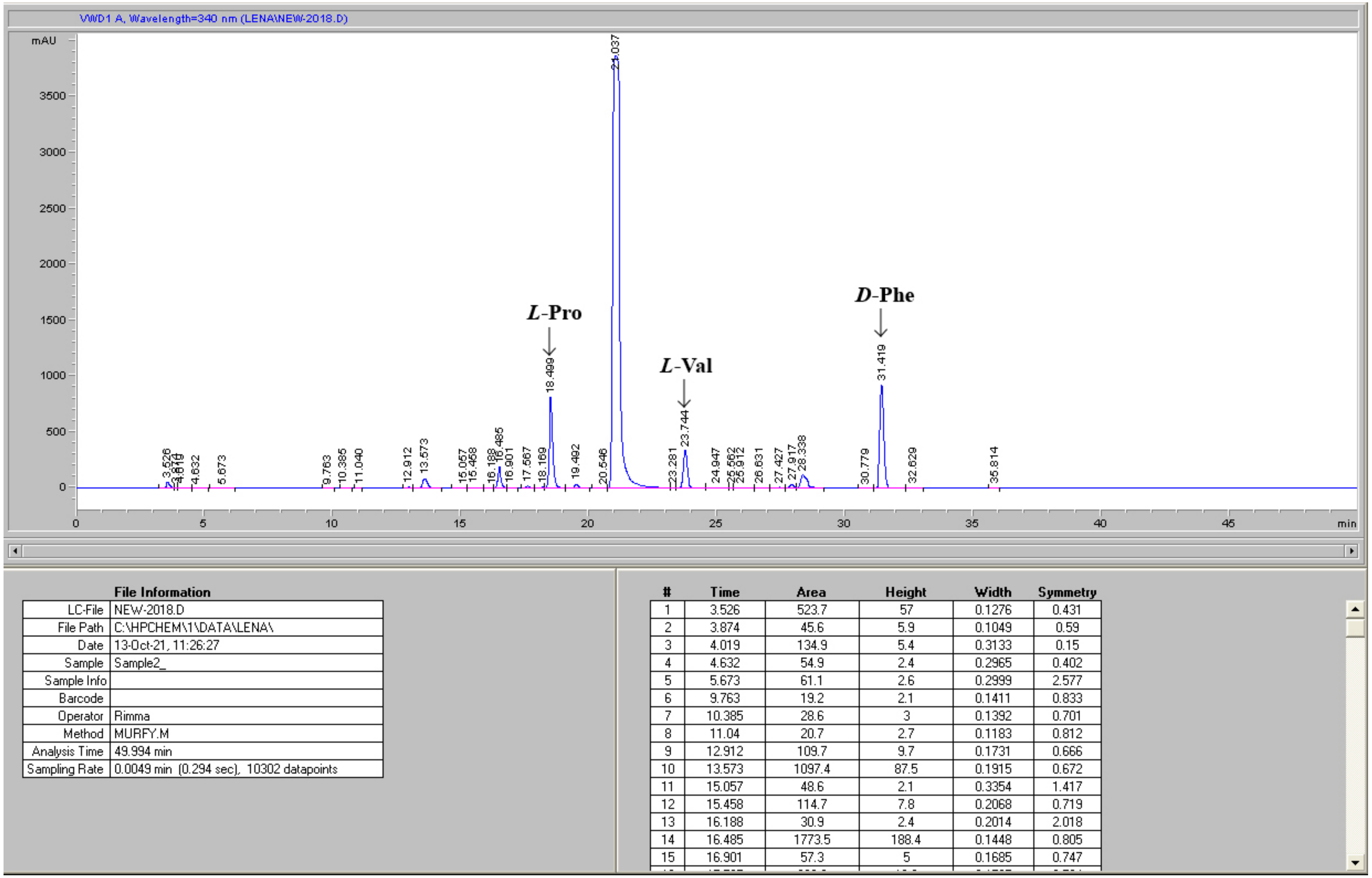

File Information

LC-File	NEW-2018.D
File Path	C:\HPCHEM\1\DATA\LENA\
Date	13-Oct-21, 11:26:27
Sample	Sample2_
Sample Info	
Barcode	
Operator	Rimma
Method	MURFY.M
Analysis Time	49.994 min
Sampling Rate	0.0049 min (0.294 sec), 10302 datapoints

#	Time	Area	Height	Width	Symmetry
1	3.526	523.7	57	0.1276	0.431
2	3.874	45.6	5.9	0.1049	0.59
3	4.019	134.9	5.4	0.3133	0.15
4	4.632	54.9	2.4	0.2965	0.402
5	5.673	61.1	2.6	0.2999	2.577
6	9.763	19.2	2.1	0.1411	0.833
7	10.385	28.6	3	0.1392	0.701
8	11.04	20.7	2.7	0.1183	0.812
9	12.912	109.7	9.7	0.1731	0.666
10	13.573	1097.4	87.5	0.1915	0.672
11	15.057	48.6	2.1	0.3354	1.417
12	15.458	114.7	7.8	0.2068	0.719
13	16.188	30.9	2.4	0.2014	2.018
14	16.485	1773.5	188.4	0.1448	0.805
15	16.901	57.3	5	0.1685	0.747
[illegible]	[illegible]	[illegible]	[illegible]	[illegible]	[illegible]

Figure S34. HPLC profiles of *L*-FDAA-derivatives of asterripeptide C (3) HP (a), asterripiptide C+*L*-Val H (b)P and asterripiptide C HP+*D*,*L*-Val (c)

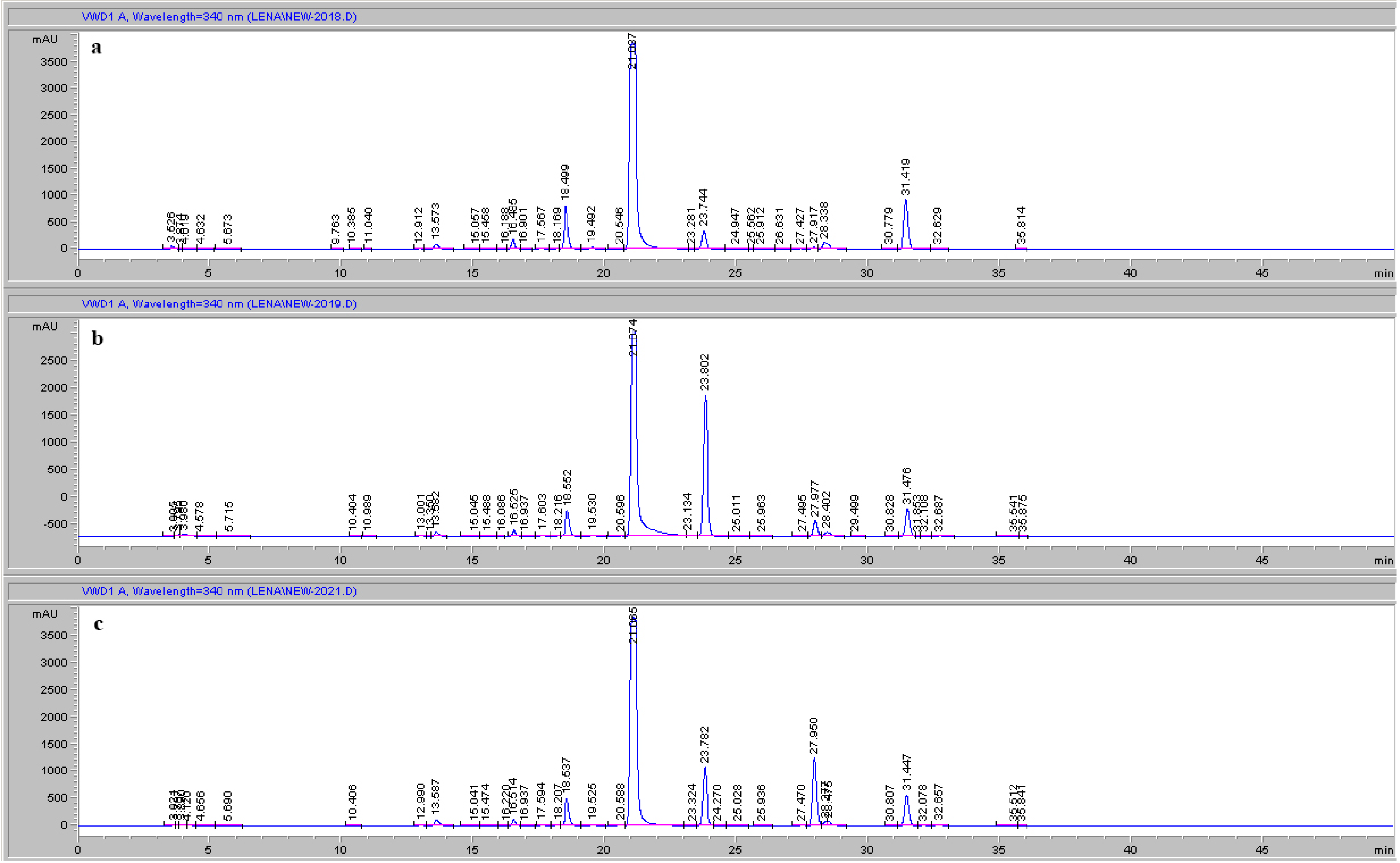

Figure S35. HPLC profiles of *L*-FDAA-derivatives of asterripeptide C (3) HP (a), asterripiptide C HP+*L*-Pro (b) and asterripiptide C HP+*D*,*L*-Pro (c)

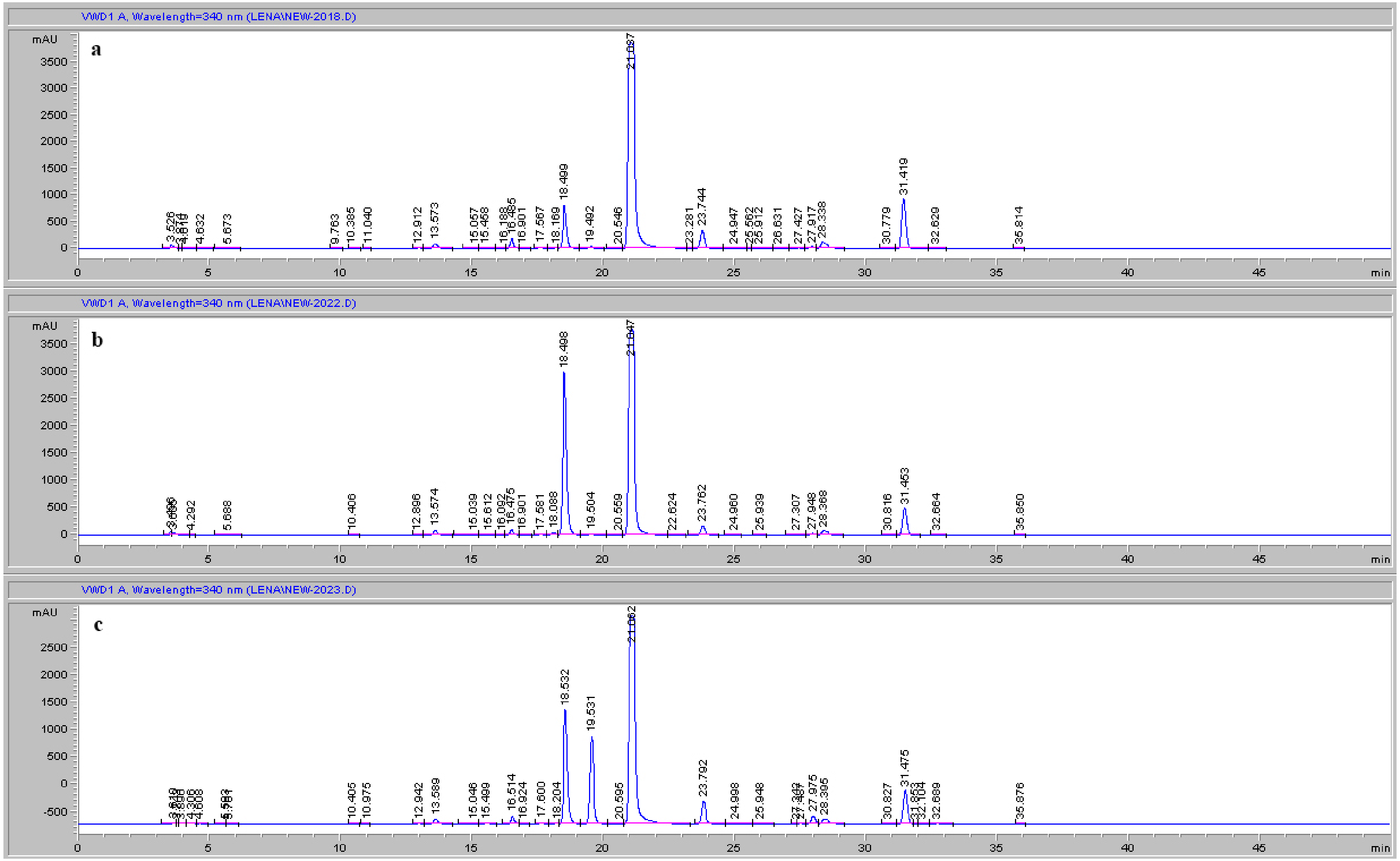

Figure S36. HPLC profiles of *L*-FDAA-derivatives of asterripeptide C (3) HP (a), asterripiptide C HP+ *D*,*L*-Phe (b) and asterripiptide C HP+*L*-Phe (c)

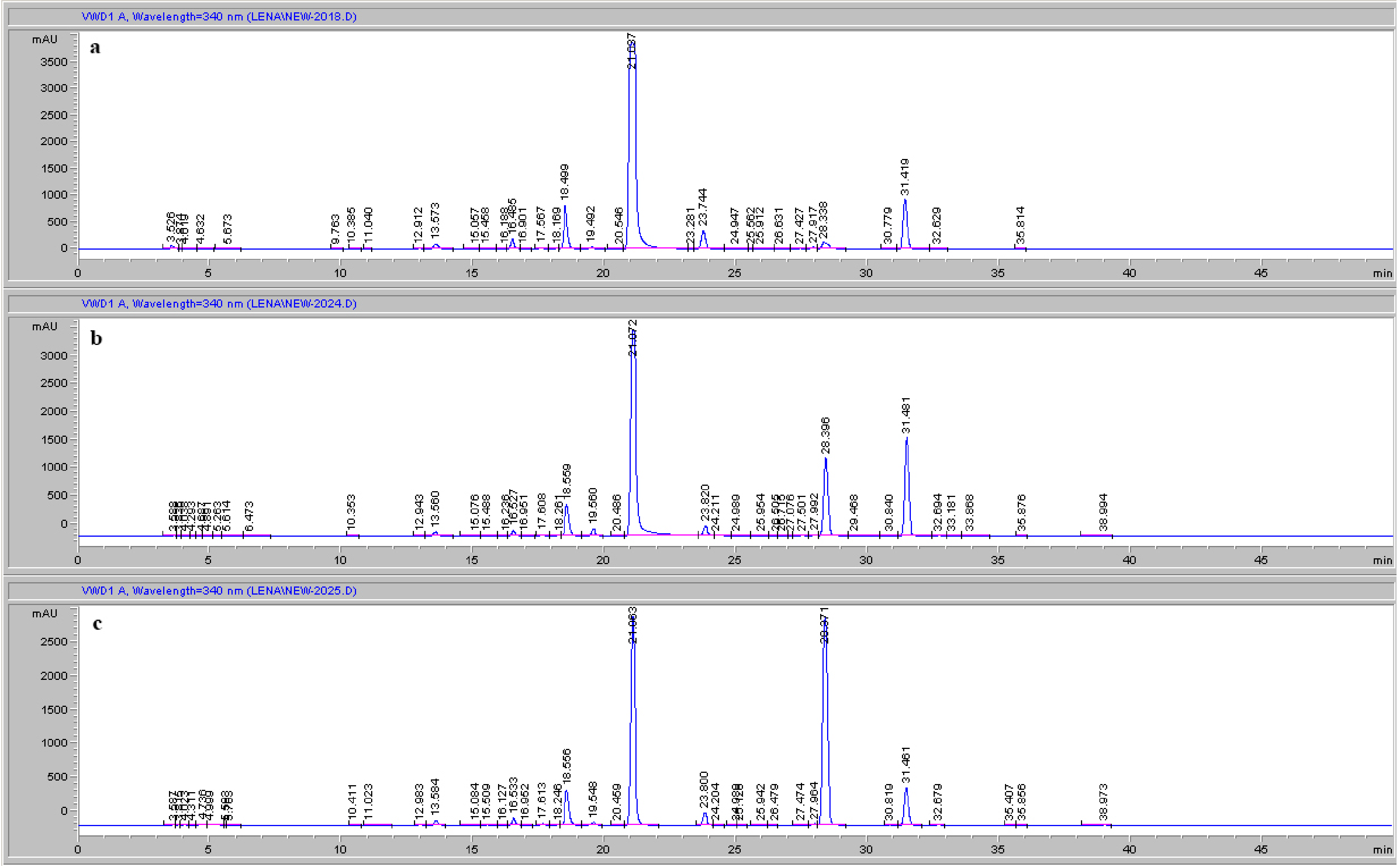

Supplement: Supplementary file 1 [file marinedrugs-20-00077-s001.zip › marinedrugs-1556162-supplementary.pdf]
